# *TaARF4* genes are linked to root growth and plant height in wheat

**DOI:** 10.1093/aob/mcy218

**Published:** 2018-12-24

**Authors:** Jingyi Wang, Ruitong Wang, Xinguo Mao, Long Li, Xiaoping Chang, Xueyong Zhang, Ruilian Jing

**Affiliations:** National Key Facility for Crop Gene Resources and Genetic Improvement, Institute of Crop Science, Chinese Academy of Agricultural Sciences, Beijing, China

**Keywords:** ABA, association analysis, auxin response factor, haplotypes, IAA, plant growth, root, *Triticum aestivum*

## Abstract

**Background and Aims:**

Auxin response factors (ARFs) as transcription activators or repressors have important roles in plant growth and development, but knowledge about the functions of wheat ARF members is limited. A novel ARF member in wheat (*Triticum aestivum*), *TaARF4*, was identified, and its protein function, haplotype geographic distribution and allelic frequencies were investigated.

**Methods:**

Tissue expression of *TaARF4* was analysed by real-time PCR. Sub-cellular localization was performed using green fluorescent protein (GFP)-tagged TaARF4. Ectopic expression of *TaARF4-A* in arabidopsis was used to study its functions. Electrophoretic mobility shift assays (EMSAs), chromatin immunoprecipitation (ChIP) analyses and gene expression were performed to detect TaARF4 target genes. A dCAPS (derived cleaved amplified polymorphic sequence) marker developed from *TaARF4-B* was used to identify haplotypes and association analysis between haplotypes and agronomic traits.

**Key Results:**

*TaARF4-A* was constitutively expressed and its protein was localized in the nucleus. Ectopic expression of *TaARF4-A* in arabidopsis caused abscisic acid (ABA) insensitivity, shorter primary root length and reduced plant height (PH). Through expression studies and ChIP assays, TaARF4-A was shown to regulate *HB33* expression which negatively responded to ABA, and reduced root length and plant height by repressing expression of *Gretchen Hagen 3* (*GH3*) genes that in turn upregulated indole-3-acetic acid content in arabidopsis. Association analysis showed that *TaARF4-B* was strongly associated with PH and root depth at the tillering, jointing and grain fill stages. Geographic distribution and allelic frequencies suggested that *TaARF4-B* haplotypes were selected in Chinese wheat breeding programmes. An amino acid change (threonine to alanine) at position 158 might be the cause of phenotype variation in accessions possessing different haplotypes.

**Conclusions:**

Ectopic expression and association analysis indicate that TaARF4 may be involved in root length and plant height determination in wheat. This work is helpful for selection of wheat genotypes with optimal root and plant architecture.

## INTRODUCTION

Auxin, a plant hormone, plays important roles in plant growth and development (Guilfoyle, 2007; Guan *et al.*, 2017; Luo *et al.*, 2018). According to a model of the auxin-mediated signalling pathway in arabidopsis (Dharmasiri *et al.*, 2005; Chapman and Estelle, 2009) at low auxin level, auxin response factor (ARF) binds to an auxin response element (AuxRE) together with Aux/IAA. Aux/IAA represses ARF activity and auxin-responsive gene expression. With high auxin concentrations, auxin receptor E3 ubiquitin ligases TIR1/AFB and Aux/IAA form a complex with auxin (Gray *et al.*, 2001; Tan *et al.*, 2007; Calderón Villalobos *et al.*, 2012). The complex initiates degradation of the Aux/IAA proteins by the 26S proteasome, then de-represses ARF activity and enables auxin-responsive gene activation (Zenser *et al.*, 2001; Kepinski and Leyser, 2005).

Previous studies demonstrated that ARFs as transcription factors could bind to TGTCTC-containing AuxREs in the promoters of auxin-responsive genes to activate or repress their expression (Ulmasov *et al*., 1997, 1999*a*; M. Zhang *et al.*, 2017). ARF proteins are conserved, and most of them consist of three domains, i.e. an N-terminal DNA-binding domain (DBD), variable middle regions as activation (AD) or repression (RD) domains, and a C-terminal domain (CTD) (Tiwari *et al.*, 2003; Korasick *et al.*, 2014). The ARF DBD is a plant-specific B3 type that binds to AuxREs. Its nuclear magnetic resonance (NMR) solution structure has also been determined (Yamasaki *et al.*, 2004). The variable middle regions determine whether it is a transcription activator or a repressor. The AD is enriched in glutamine (Q), serine (S) and leucine (L), whereas the RD is enriched in serine (S), proline (P), leucine (L) and/or glycine (G) (Chandler, 2016; Nanao *et al.*, 2014). The CTD is a dimerization domain for combining with ARFs or Aux/IAA proteins (Kim *et al.*, 1997; Ulmasov *et al.*, 1999*b*).

The ARF family in arabidopsis contains 22 members and one pseudogene (*ARF23*) (Guilfoyle and Hagen, 2007; Remington *et al.*, 2004), and rice contains 25 ARF members (Wang *et al.*, 2007; Zhang *et al.*, 2018). The functions of ARFs in arabidopsis were revealed mainly by study of *arf* mutants. AtARF2 has roles in seed size, fertility, senescence and hormone cross-talk (Okushima *et al.*, 2005; Promchuea *et al.*, 2017). AtARF3 functions in pattern development of floral meristems and reproductive organs (Sessions *et al.*, 1997), while AtARF5 works in embryo axis formation and vascular tissue development (Hardtke and Berleth, 1998). AtARF7 is involved in aerial tissue growth (Harper *et al.*, 2000), AtARF8 functions in fruit initiation (Goetz *et al.*, 2006) and AtARF19 is active in hormone cross-talk (Li *et al.*, 2006). Some AtARFs have redundant roles in plant development (Hardtke *et al.*, 2004). However, research into ARF family members in wheat is lacking.

Wheat is a staple food crop worldwide, and its root and plant architectures strongly affect grain yield (Li *et al.*, 2016). As a hexaploid species, wheat has a large and complex genome (AABBDD) that poses a challenge for gene discovery and determining gene function (International Wheat Genome Sequencing Consortium, 2014; Luo *et al.*, 2017; Zhao *et al.*, 2017; Appels *et al.*, 2018; Ling *et al.*, 2018). Association analysis is an efficient approach to reveal relationships between genes and traits (Bradbury *et al.*, 2007; Wang *et al.*, 2016). Pyramiding elite alleles through marker-assisted selection (MAS) greatly enhances the efficiency of wheat breeding (Collard and Mackill, 2008). Thus, finding elite alleles and developing functional markers are fundamental to genetic improvement of the crop.

In this study, three copies of *TaARF4* were isolated from wheat and characterized. Ectopic expression of *TaARF4* in arabidopsis resulted in reduced *HB33* expression and response to abscisic acid (ABA). *TaARF4*-overexpressiong plants also had shorter primary roots and reduced plant height; it is proposed that this is due to repressing *Gretchen Hagen 3* (*GH3*) gene expression that mediates indole-3-acetic acid (IAA) homeostasis. Association analysis showed that *TaARF4-B* was strongly associated with plant height (PH) and root depth of wheat. The geographic distribution and allelic frequencies demonstrated that *TaARF4-B* haplotypes were selected in the history of Chinese wheat breeding.

## MATERIALS AND METHODS

### Identification of *TaARF4* and construction of a phylogenetic tree

Using the URGI website tBLASTn program and arabidopsis ARF2 N-terminal DBD as a query sequence, we isolated an *ARF* member (named *TaARF4*) with cDNA (AK335756.1) and protein (CDM85878.1) sequence accession numbers in GenBank; nothing was known about its function. The full-length cDNAs of the homoeologous genes *TaARF4-A*, *TaARF4-B* and *TaARF4-D* were cloned by reverse transcription–PCR (RT–PCR) from wheat cultivar Hanxuan 10. ARF family members were identified from different plant species by a protein BLAST search in the NCBI database. The full-length sequence was used to construct a phylogenetic tree by the maximum likelihood method through the Molecular Evolutionary Genetics Analysis (MEGA) software version 5.2.

### Expression pattern analysis of *TaARF4* in wheat

Hanxuan 10, a drought- and heat-tolerant common wheat cultivar, was used for genomic sequence isolation of *TaARF4* and gene expression pattern analysis. Root and leaf tissues from 2-week-old seedlings and different tissues at the flowering stage (stem, leaf and spike, and root tissues from different depths) were sampled for spatio-temporal expression pattern analysis. Two-week-old wheat seedlings were sprayed with 50 μm ABA solution, then whole plants were sampled for ABA-induced expression analysis at 0, 1, 3, 6, 12, 24 and 48 h after treatment. Total RNAs were extracted using Trizol reagent (Invitrogen, 15596-018). cDNA was synthesized with a reverse transcription kit (TIANGEN, KR104). SYBR Premix *Ex Taq* (TaKaRa, DRR820A) was used for real-time PCR on an ABI QuantStudio^®^ 7 Flex analyser according to the manufacturer’s instructions. Because the nucleotide sequences of *TaARF4-A*, *TaARF4-B* and *TaARF4-D* were highly conserved, it was difficult to distinguish them; therefore, a common primer pair for all three copies was designed by Primer Premier 5 software (Supplementary Data Table S1). Glyceraldehyde phosphate dehydrogenase (*GAPDH*) was used as the internal control. Three biological replicates and three technical replicates were assayed for each sample.

### Sub-cellular localization of TaARF4 protein and production of TaARF4–GFP transgenic lines

The full-length cDNA of *TaARF4-A* was cloned into the modified vector pCAMBIA1300 at the *Xba*I and *Spe*I sites under control of the 35S promoter and with a green fluorescent protein (GFP) tag. Primers are listed in Supplementary Data Table S2. The constructs and empty vectors were separately transfected into *Agrobacterium tumefaciens* strain GV3101 by electroporation. For transient expression, *Agrobacterium* was cultured in 5 mL of YEB medium supplemented with 100 mg mL^–1^ rifampicin, 50 mg mL^–1^ kanamycin then infiltrated into the abaxial side of 5-week-old *Nicotiana benthamiana* leaves. The *Agrobacterium*-infected tobacco plants were grown in a greenhouse for 3 d before GFP fluorescence in leaves was observed with a confocal laser scanning microscope (Zeiss LSM700). At the same time, stable transgenic arabidopsis lines were produced by infection with *Agrobacterium* by the floral dip method (Clough and Bent, 1998). Transformants were selected on agar plates containing 50 μg mL^–1^ hygromycin B. GFP fluorescence in homozygous T_3_ generation plants was detected in 1-week-old transgenic plant roots by a Zeiss LSM700 microscope, and DAPI (4’,6-diamidino-2-phenylindole) was used to stain nuclei.

### Purification of TaARF4-Δ protein and electrophoretic mobility shift assays (EMSAs)

The TaARF4-A N-terminus containing the DBD (TaARF4-Δ, amino acids 1–350) was fused with a glutathione *S*-transferase (GST) tag and expressed in *Escherichia coli* BL21 cells. The recombinant protein was induced by 0.2 mm isopropyl-β-d-thiogalactoside (IPTG), and the *E. coli* were incubated at 18 °C for 12 h. TaARF4-Δ protein was purified by glutathione–Sepharose 4B (GE Healthcare, 52-2303-00). P3 containing an inverted repeat of AuxRE was used as a probe. Cold (unlabelled) P3, cold mutated P3, biotin-labelled P3, biotin-labelled mutated P3 and their reverse complementary sequences were synthesized and annealed. EMSA was performed using a LightShift^®^ Chemiluminescent EMSA Kit (Thermo Scientific, 20148) according to the manufacturer’s instructions. Briefly, probes and protein in binding buffer were incubated at room temperature for 20 min. The binding reaction mixes were separated in 5 % native polyacrylamide gels. DNA fragments in the gels were transferred to nitrocellulose membranes. After cross-linking, the membranes were incubated in blocking buffer, and then transferred to conjugate/blocking buffer. After washing, membranes were incubated in Substrate Equilibration Buffer, followed by Substrate Working Solution (Revzin, 1989). Finally, the membranes were photographed with a CCD camera.

### Phenotypic analyses of transgenic arabidopsis

#### ABA sensitivity in seed germination.

Seeds of the wild type (Columbia, Col) and transgenic lines were sterilized and placed on Murashige and Skoog (MS) medium (Sigma-Aldrich, M5519) and MS medium supplemented with 0.5 μm ABA. After incubation for 2 d at 4 °C, the plates were transferred to a growth chamber with a 23 h light (21 °C)/1 h darkness (19 °C) cycle. Numbers of seedlings with cotyledon greening were scored after cultivation for 1 week.

#### Root length and plant height.

Seeds for root growth assays were sown on MS plates and grown in a chamber with 23 h light (21 °C)/1 h darkness (19 °C) for 5 d. Seedlings were then transferred to MS medium vertical culture with root tips placed at the same level. Photographs were taken after 7 d, and new growth parts of the primary roots were measured and analysed by Image J software. The growth rate for each genotype was calculated. At least 15 plants from three Petri dishes were measured for each experiment, and three independent biological experiments were performed. Two-month-old seedlings growing in a forest soil:vermiculite (1:1) mixture with 16 h light (21 °C)/8 h darkness (19 °C) in a greenhouse were used to assess plant height. At least 15 seedlings were measured from three pots for each experiment, and three independent biological experiments were performed.

### Detection of gene expression

Total RNAs were extracted from 2-week-old Columbia and transgenic arabidopsis; cDNA synthesis and real-time PCR were performed as mentioned above. *HB33*, *GH3.2* and *GH3.5* gene expression patterns were determined using *Tubulin* as the control. Primers are listed in Supplementary Data Table S1.

### Chromatin immunoprecipitation (ChIP) analyses

Chromatin immunoprecipitation was carried out on 2 g of 2-week-old transgenic arabidopsis plants (GFP and TaARF4–GFP) according to the method of Saleh *et al.* (2008). Briefly, after DNA and protein were cross-linked with 1 % formaldehyde, chromatin was isolated, and DNA was sheared on ice by sonicating five times for 15 s at 1 min intervals using an LTRASONIC PROCESSOR-500. Salmon sperm DNA/protein A agarose (Millipore, 16-157) and anti-GFP antibody (Abcam, AB290) were used to precipitate the DNA and protein complex. Then DNA and protein were reverse cross-linked, and DNA was precipitated. The precipitates were separately dissolved in 500 μL of TE to carry out real-time PCR using 5 μL of ChIP product for each one. Three independent biological experiments, each with three technical replicates, were performed. Primers are shown in Supplementary Data Table S3.

### Free IAA extraction and analysis

Three independent replicates were performed. For each replicate, about 200 mg of 2-week-old fresh seedling tissue were ground in liquid nitrogen, weighed and IAA was extracted at –20 °C for 24 h with 2 mL of cold methanol. Antioxidant and internal standard (^2^H_2_-IAA, CDN Isotopes) were added and samples were purified using an Oasis MAX solid-phase extract cartridge (150 mg 6 mL^–1^; Waters), and analysed by a UPLC-MS/MS system (ACQUITY UPLC; Waters and Quattro Premier XE; Waters) as previously described (Wang *et al.*, 2015). This part of the work was conducted on a plant hormone platform at the Institute of Genetics and Developmental Biology, Chinese Academy of Sciences.

### Gene sequence polymorphism and functional marker development

Thirty-two wheat accessions with wide variation identified by simple sequence repeat (SSR) markers were chosen to sequence *TaARF4* fragments for analysis of polymorphisms. Genome-specific primers were designed for amplification of *TaARF4-A*, *TaARF4-B* and *TaARF4-D* fragments from the A, B and D genomes (Supplementary Data Table S4). Purified PCR products were ligated into pEASY-Blunt vectors and transformed into *E. coli* top 10 competent cells. Positive clones were selected and sequenced with an ABI 3730 DNA Analyzer. Sequence polymorphism was analysed by SeqMan software. Derived cleaved amplified polymorphic sequence (dCAPS) primers for target genes were developed based on dCAPS Finder 2.0 software (http://helix.wustl.edu/dcaps/dcaps.html). The genotypes of the wheat accessions were identified as follows: first, *TaARF4* genes in A, B and D genomes were amplified using genome-specific primers; second, 1 μL of PCR product as template was subjected to a second round of PCR; third, the second-round PCR products were digested by restriction endonucleases and electrophoresed in 4 % agarose gels.

### Wheat populations used in the study

Five wheat germplasm populations were used for different research purposes. Accession names in the populations are listed in Supplementary Data Table S5. Population 1 consisted of 150 doubled haploid (DH) lines derived from the cross Hanxuan 10 × Lumai 14. This population was employed for gene mapping and analysis of the effects of different haplotypes. Population 2 consisted of 262 accessions mainly from the Northern Winter Wheat Zone and Yellow and Huai River Valleys Facultative Wheat Zone, and was used for association analysis between agronomic traits and genotypes. Population 3 consisted of 323 accessions from the same region and was used for association analysis between root traits and haplotypes. Population 4 consisting of 157 landraces and Population 5 consisting of 348 modern cultivars from all of ten major wheat zones of China were used to determine the temporal and spatial distributions of haplotypes.

### Agronomic and root traits

Population 1 and Population 2 were planted at Shunyi (40°23′N, 116°56′E) and Changping (40°13′N, 116°13′E), Beijing, over 3 years (2010–2012) for measurement of plant height under two water regimes, drought-stressed (DS) and well-watered (WW). The amounts of rainfall during the three growing seasons were 131, 180 and 158 mm, respectively. DS plots were rain-fed, while WW plots were irrigated with 750 m^3^ ha^–1^ (75 mm) at the pre-overwintering, booting, flowering and grain fill stages when the amounts of rainfall were insufficient during each corresponding period. A greenhouse covered with polythene at the flowering stage to increase temperature and simulate heat stress (HS) was used at Shunyi. Seven agronomic traits, i.e. PH, length of penultimate internode (LPI), spike length (SL), 1000 grain weight (TGW), number of spikes per plant (NSP), number of spikelets per spike (NSS) and numbers of grain per spike (NGS), were measured under both water regimes, with and without heat stress. Population 3 was planted in PVC tubes, and root phenotypes were recorded at the seedling, tillering, jointing and grain fill stages. Population 5 for investigation of agronomic traits was planted at Luoyang (34°61′N, 112°45′E) in Henan province in 2002 and 2005, and at Shunyi, Beijing in 2010.

### Association analysis

Population structure was examined by software Structure v2.3.2 (B. Zhang *et al.*, 2017). Association analysis was performed by the mixed linear model (MLM) in TASSEL 5, in which population structure parameter Q was used. Associations at *P* < 0.05 were considered significant. Statistical analyses were conducted using SPSS 16.0 software.

### Phosphorylated site prediction

TaARF4 protein sequences were submitted to the NetPhos 3.1 Server website (http://www.cbs.dtu.dk/services/NetPhos/). Serine, threonine or tyrosine phosphorylation sites were predicted, and both generic and kinase-specific predictions were performed.

## RESULTS

### Cloning and structural analysis of *TaARF4* genes

Three full-length cDNA sequences of *TaARF4* were cloned from wheat cultivar Hanxuan 10, and named *TaARF4-A*, *TaARF4-B* and *TaARF4-D* according to their genomic origins. The three *TaARF4* genes were highly similar in sequence, encoding 797, 796 and 796 amino acids, respectively, and very similar in protein structure identity at 99.08 %. Like other ARF family members, TaARF4s consisted of a DBD at the N-terminus, an RD that is usually enriched in serine (S), proline (P), leucine (L) and/or glycine (G) (SPL-RD) in the middle region, and a CTD (Supplementary Data Fig. S1A). The phylogenetic tree (Supplementary Data Fig. S1B) showed that the TaARF4s were more similar to ARF4s in monocotyledons than those in arabidopsis.

### Expression pattern of *TaARF4* in wheat tissues

**Fig. 1.**
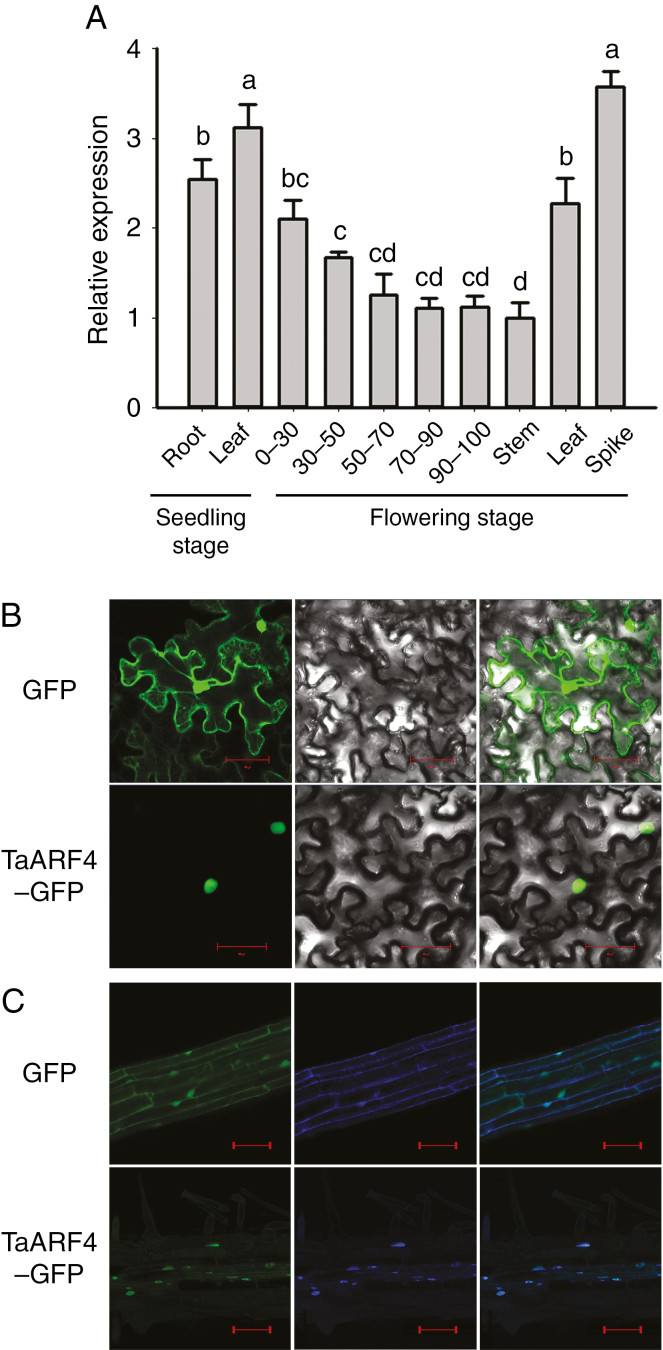
Expression patterns of *TaARF4* and sub-cellular localization of TaARF4 protein. (A) Tissue expression patterns of *TaARF4* were detected by real-time PCR. *GAPDH* was used as the control. Three independent biological experiments, each with three technical replicates, were carried out. Error bars are 2 × s.e. ‘0–30’ indicates the root section from the ground to 30 cm depth at flowering stage; ‘30–50’ indicates the root section from 30 to 50 cm; ‘50–70’ indicates the root section from 50 to 70 cm; ‘70–90’ indicates the root section from 70 to 90 cm; and ‘90–100’ indicates the root section from 90 to 100 cm. Significant differences were calculated based on ANOVA. (B) GFP and TaARF4–GFP were transiently expressed in transfected tobacco leaf cells. Green fluorescence images are on the left, bright field images are in the middle and merged images are on the right. Scale bars = 20 μm. (C) GFP and TaARF4–GFP were stably expressed in transgenic arabidopsis roots. Green fluorescence images are on the left, DAPI-stained images are in the middle and merged images are on the right. Scale bars = 100 μm.

Real-time PCR was performed to identify expression patterns of the *TaARF4* genes. *TaARF4* was constitutively expressed in wheat tissues (roots and leaves of seedlings; roots, stems, leaves and spikes at flowering). The highest expression level was detected in the spikes (Fig. 1A), and in roots expression levels were lower at greater depths. Constitutive expression of *TaARF4* genes suggested that they might have roles in growth of all tissues.

### Subcellular localization of TaARF4 protein

Fluorescence was detected in all cell parts of tobacco epidermal leaf cells and root cells of T_3_ transgenic arabidopsis lines with the empty vector carrying the *GFP* gene, whereas TaARF4–GFP was specifically distributed in the nucleus (Fig. 1B, C).

### TaARF4 protein binds to the AuxRE *cis*-element *in vitro*

**Fig. 2.**
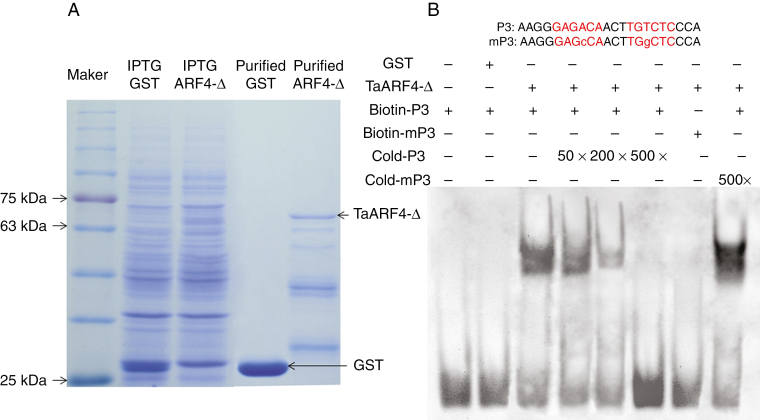
Expression and purification of TaARF4-Δ protein and electrophoretic mobility shift assays (EMSAs). (A) Expression and purification of TaARF4-Δ protein in *E. coli* strain BL21. Purified GST and TaARF4-Δ protein are marked by arrows. Lane 1 is a pre-stained protein marker. Lane 2 contains the entire *E. coli* protein suspension that was transfected by an empty pGEX-4T1 vector after IPTG induction. Lane 3 contains an entire *E. coli* protein suspension, which was transfected by pGEX-4T1 vector fused with *TaARF4-Δ* after IPTG induction. Lane 4 contains purified GST protein, and lane 5 contains purified TaARF4-Δ. (B) EMSA of purified TaARF4-Δ protein and P3 probe. Probe P3 and mutant P3 (mP3) sequences are shown above the lanes. The *cis*-element regions are shown in red script. Lower case letters indicate mutated bases. Lane 1 contains only biotin-labelled P3 probe. Lane 2 contains biotin-labelled P3 probe and GST protein. Lane 3 contains biotin-labelled P3 probe and TaARF4-Δ protein. Lane 4 contains biotin-labelled P3 probe and TaARF4-Δ protein with 50× cold (unlabelled) P3 probe. Lane 5 contains biotin-labelled P3 probe and TaARF4-Δ protein with 200× cold P3 probe. Lane 6 contains biotin-labelled P3 probe and TaARF4-Δ protein with 500× cold P3 probe. Lane 7 contains biotin-labelled mP3 probe and TaARF4-Δ protein. Lane 8 contains biotin-labelled P3 probe and TaARF4-Δ protein with 500× cold mP3 probe. Upper bands show TaARF4-Δ protein bound to biotin-labelled P3 probe; lower bands show free probe.

The promoters of auxin response genes share a consensus sequence (TGTCTC) known as the AuxRE. EMSAs were performed to test whether TaARF4 binds to AuxRE *cis*-acting elements *in vitro*. Since we could not purify the entire TaARF4 protein, the TaARF4 N-terminus with the DBD (TaARF4-Δ, amino acids 1–350) of the corresponding cDNA was cloned into a pGEX-4T1 vector (GST tag). Expression and purification of protein were carried out using *E. coli* BL21 cultures (Fig. 2A). A DNA-binding band detected with addition of TaARF4-Δ and biotin-labelled P3 probes had slower migration compared with the free probe (Fig. 2B, lane 3), whereas TaARF4-Δ could not bind to biotin-labelled mutated P3 probes (Fig. 2B, lane 7), and no target band was visible in the GST control (Fig. 2B, lane 2). Along with the gradual increase in cold P3 probe concentrations, the biotin-labelled DNA-binding band was diminished (Fig. 2B, lanes 4–6). The cold mutated P3 (mP3) probe did not compete with labelled P3 probes (Fig. 2B, lane 8). These results showed that the TaARF4 N-terminal DBD binds to AuxRE *in vitro*.

### Overexpression of *TaARF4* enhances ABA resistance in arabidopsis

**Fig. 3.**
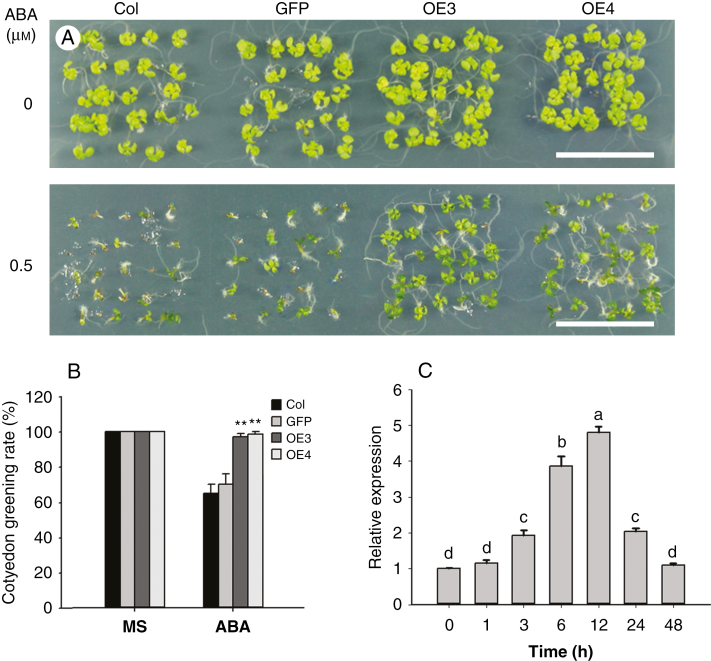
Overexpression of *TaARF4* enhances arabidopsis resistance to ABA. (A) Cotyledon greening of Columbia (Col), GFP and two *TaARF4-OE* lines on MS or MS supplemented with 0.5 μm ABA. After sterilization, seeds were imbibed on MS medium plates with or without ABA and cultured in a growth chamber for 7 d. Scale bar = 2 cm. (B) Statistical analyses of cotyledon greening rates. There were three independent experiments, each with three replicates (in different plates), and in each replicate at least 25 seeds were counted. Error bars are 2× s.e. *P < 0.05, **P < 0.01 (Student’s *t*-test). (C) Expression patterns of *TaARF4* following ABA treatment were detected by real-time PCR. Two-week-old wheat seedlings were sprayed with 50 μm ABA solution, and whole plants were harvested at 0, 1, 3, 6, 12, 24 and 48 h after treatment. *GAPDH* was used as the control. Three independent biological experiments, each with three technical replicates, were carried out. Error bars are 2 × s.e. Significant differences were calculated based on ANOVA.

*TaARF4-A*, *TaARF4-B* and *TaARF4-D* have highly similar sequences, therefore *TaARF4-A* was chosen to represent all three *TaARF4* genes for characterization of functions. *TaARF4-A* in a vector in which its expression was driven by the 35S promoter was transformed into arabidopsis. The Col and empty vector line (GFP) were used as controls. All six transgenic lines showed higher *TaARF4* expression levels than the controls (Supplementary Data Fig. S2). We selected transgenic lines OE3 and OE4 to investigate stay green in cotyledons in response to ABA. Under normal conditions (MS medium), there was no significant difference between the controls and *TaARF4* overexpression lines. Following culture on MS medium with 0.5 μm ABA for 7 d, the cotyledon greening ratio was about 67 % for the controls, compared with about 97 % for the two overexpression lines (Fig. 3A, B). We also detected the expression level of *TaARF4* under ABA treatment. As shown in Fig. 3C, the transcription level of *TaARF4* was upregulated by ABA and reached its highest level on exposure to ABA for 12 h, a level which was about 5-fold that of the non-treated control. At 48 h, the expression level declined to a similar level to that of the control. These results indicated that overexpression of *TaARF4* led to resistance to ABA, and that *TaARF4* negatively regulates ABA response in arabidopsis.

### TaARF4 takes part in ABA response by binding to the HB33 promoter and regulating its expression

**Fig. 4.**
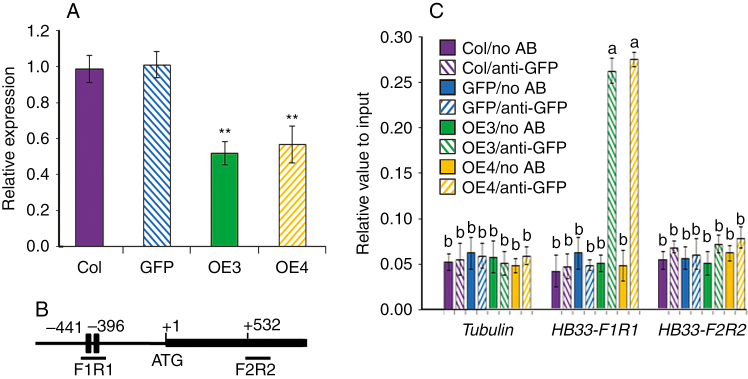
*HB33* expression in *TaARF4-OE* plants and ChIP assay on the promoter of *HB33*. (A) Expression of *HB33* was negatively regulated by TaARF4. Expression of *HB33* was detected in 2-week-old seedlings of Col, GFP and two *TaARF4-OE* plants. Three biologically independent experiments, each with three technical replicates, were performed. Error bars are 2 × s.e. *P < 0.05, **P < 0.01 (Student’s *t*-test). (B) Bars indicate putative TaARF4-binding sites of *HB33*. The ATG translation start site is indicated at position +1. Primers used for ChIP PCR are listed in Supplementary Data Table 3. (C) ChIP assay of TaARF4 binding to the promoter of *HB33*. Col and empty GFP seedlings were used as negative controls. Two transgenic lines (*TaARF4-GFP-OE3* and *TaARF4-GFP-OE4*) and GFP antibody were used for the ChIP assay. Real-time PCR was carried out to show DNA abundance relative to input. Three biological replicates were performed, and each biological replicate had three technical replicates. Error bars are 2 × s.e. Significant differences were calculated based on ANOVA.

It was reported that lots of homeobox gene family members took part in abiotic stress response and one member HB33 had an important role in ABA response (Söderman *et al.*, 1996; Tan and Irish, 2006; Wang *et al.*, 2011; Bhattacharjee *et al.*, 2016). Therefore, we analysed *HB33* expression in controls and *TaARF4-OE* transgenic plants. Expression was lower in *TaARF4-OE* plants than in the controls (Fig. 4A). There are two AuxREs in the *HB33* promoter region, one at –441 bp in the forward direction and the other in the reverse direction at –396 bp (Fig. 4B). ChIP assays were carried out to determine whether TaARF4 could directly bind to the *HB33* promoter regions *in vivo*. GFP antibody was used for precipitations of protein and DNA. DNA abundance relative to the input was detected by real-time PCR. As shown in Fig. 4C, TaARF4–GFP bound to the *HB33* promoter region, where there are two AuxRE *cis*-elements (*HB33-F1R1*), but could not bind to the *Tubulin* gene promoter region and *HB33* coding region (*HB33-F2R2*) that lack AuxRE. These results indicated that TaARF4 can take part in the ABA pathway by binding to the *HB33* promoter region to regulate *HB33* expression.

### Overexpression of *TaARF4* in arabidopsis led to shorter primary roots and plant height

**Fig. 5.**
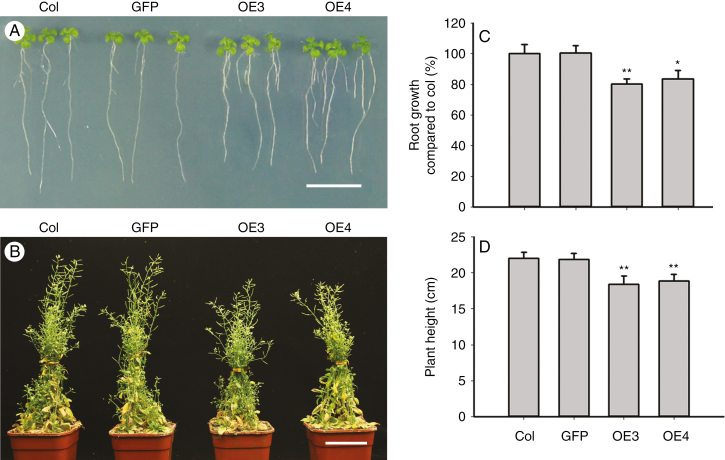
Overexpression of *TaARF4* in arabidopsis changes root architecture and plant height. (A) Overexpression of *TaARF4* produces shorter primary roots and longer lateral roots than the control. Five-day-old seedlings were transferred to vertical culture and photographed after 7 d. Scale bar = 1 cm. (B) Overexpression of *TaARF4* in arabidopsis caused shorter plant height than the control. Two-month-old plants growing in soil were used to compare plant heights. (C) Relative primary root growth of seedlings of GFP, OE3 and OE4 compared with Col. Three independent experiments were performed and at least 20 seedlings were measured in each experiment. Scale bar = 5 cm. Error bars are 2 × s.e. (D) Statistical analyses of plant height. Three independent experiments; in each experiment at least 20 plants were counted. Error bars are 2 × s.e. *P < 0.05, **P < 0.01 (Student’s *t*-test).

Since many *arf* mutants were reported to affect plant development, we investigated the morphological characteristics of *TaARF4*-overexpressing arabidopsis plants. When 5-day-old seedlings overexpressing *TaARF4* were transferred to MS medium in vertical orientation for 7 d, they developed shorter primary roots and longer lateral roots than Col and transgenic GFP controls (Fig. 5A). Primary root growth of the transgenic lines was reduced to about 82 % of that of the controls (Fig. 5C). The PHs of 2-month-old transgenic lines planted in soil were about 85 % of that of the controls (Fig. 5B, D). Collectively, these data indicate that TaARF4 has roles in root growth and PH.

### TaARF4 binds to the *GH3* promoters, and regulates *GH3* gene expression and IAA homeostasis in arabidopsis

**Fig. 6.**
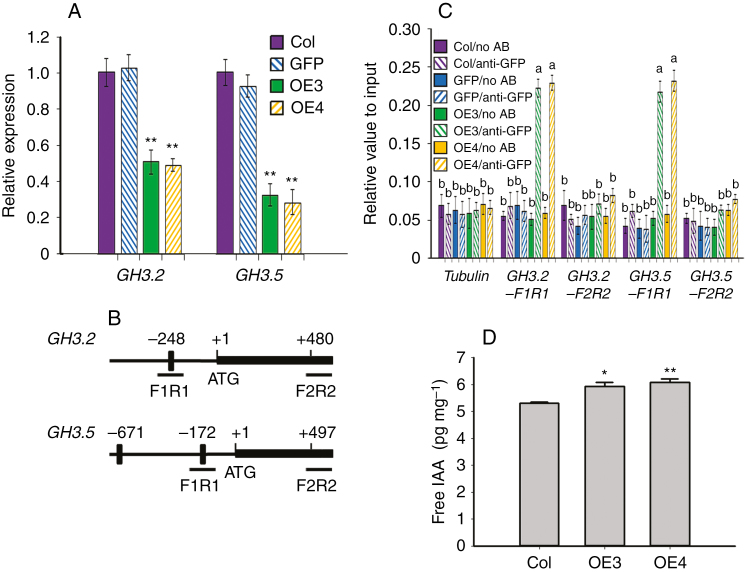
*GH3* gene expression in *TaARF4-OE* plants and ChIP assay on the promoters of *GH3* genes. (A) Expression of *GH3* genes was negatively regulated by *TaARF4*. Expression of *GH3.2* and *GH3.5* was detected in 2-week-old seedlings of Col, GFP and two *TaARF4-OE* plants. Three biologically independent experiments, each with three technical replicates, were performed. Error bars are 2 × s.e. *P < 0.05, **P < 0.01 (Student’s *t*-test). (B) Bars indicate putative TaARF4-binding sites of *GH3* genes. The ATG translation start site is indicated at position +1. Primers used for ChIP PCR are listed in Supplementary Data Table 3. (C) ChIP assay of TaARF4 binding to the promoter of *GH3* genes. Col and empty GFP seedlings were used as the negative control. Two transgenic lines (*TaARF4-GFP-OE3* and *TaARF4-GFP-OE4*) and GFP antibody were used for the ChIP assay. Real-time PCR was carried out to show DNA abundance relative to input. Three biological replicates were performed, and each biological replicate had three technical replicates. Error bars are 2 × s.e. Significant differences were calculated based on ANOVA. (D) Free IAA levels in 2-week-old seedlings. Three independent replicates were performed. Error bars are 2 × s.e. *P < 0.05, **P < 0.01 (Student’s *t*-test).

Three kinds of genes respond to auxin, namely *Aux*/*IAA*, *SAUR* and *GH3* (Nemhauser *et al.*, 2006). The *GH3* gene was first discovered as an auxin response gene in *Glycine max* (Hagen *et al.*, 1984). It was shown to conjugate various amino acids to jasmonic acid (JA) and auxin, facilitating hormone activation, storage or transport, and helping to maintain hormone homeostasis (Staswick *et al.*, 2005). However, the relationship of TaARF4 and GH3 is not clear. We therefore checked *GH3* gene expression and IAA homeostasis of *TaARF4*-overexpressing arabidopsis plants. Expression levels of *GH3.2* and *GH3.5* were lower in *TaARF4-OE* plants compared with the controls (Fig. 6A). In the *GH3.2* promoter region there is one AuxRE at –248 bp; and in the *GH3.5* promoter region, there are two AuxREs at –172 bp and –671 bp (Fig. 6B). ChIP assays using GFP antibody were carried out to determine whether TaARF4 could directly bind to the *GH3* promoter regions *in vivo*. Real-time PCR results indicated that TaARF4–GFP bound to the AuxRE regions, not the coding regions. In the absence of the GFP antibody, we could not detect TaARF4–GFP bound to DNA fragments (Fig. 6C). Free IAA contents of OE3 and OE4 were 12.1 and 14.9 % higher than in the Col wild type (Fig. 6D). Thus, it is proposed that TaARF4 can bind to the promoter region of *GH3* genes to repress *GH3* gene expression, and further repress free IAA changing into other forms, leading to higher free IAA content in OE plants. This suggests that the shorter primary root length and reduced height of *TaARF4*-overexpressing arabidopsis plants might be due to higher free IAA content, which inhibits apical dominance.

### Sequence polymorphism and genetic mapping

**Fig. 7.**
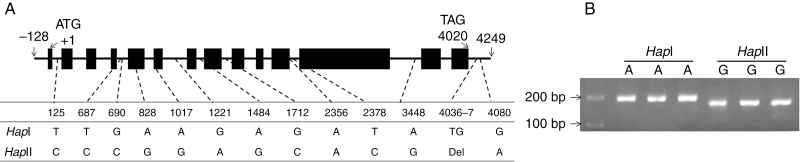
Nucleotide polymorphisms and functional marker development for *TaARF4-B*. (A) Twelve single nucleotide polymorphisms and one deletion were detected in the *TaARF4-B* genomic region. Black rectangles indicate exons. (B) A dCAPS marker was developed based on SNP-828 (A/G). Using *Sac*I restriction endonuclease, a 192 bp PCR product amplified from accessions with SNP-828G (*Hap*II) can be digested to 168 and 24 bp, whereas the 192 bp PCR product amplified from accessions with SNP-828A (*Hap*I) could not be digested. A 100 bp DNA ladder is shown on the left.

Genome-specific primers were designed based on the polymorphism detected in the flanking regions of *TaARF4* genes in three genomes. Using these primers, *TaARF4-A*, *TaARF4-B* and *TaARF4-D* genomic fragments were separately cloned from A, B and D genomes; their lengths were 4584, 4375 and 4123 bp, respectively. We sequenced genomic fragments from 32 diverse accessions, and no nucleotide polymorphism was detected in *TaARF4-A* or *TaARF4-D*. However 13 variants of *TaARF4-B* formed two haplotypes (Fig. 7A). Among the variants, only one single nucleotide polymorphism [SNP (A/G)] at 1017 bp resulted in an amino acid change (threonine to alanine). A dCAPS marker was developed based on an SNP (A/G) at 828 bp (Fig. 7B). The dCAPS marker was then used to genotype Population 1 (150 DH lines). *TaARF4-B* was mapped on chromosome 3B and was flanked by markers *P3622.4* and *P2076* (Supplementary Data Fig. S3A). One quantitative trait locus (QTL) affecting PH was earlier mapped in this marker interval (Wu *et al.*, 2010). The genetic distances between two markers and *TaARF4-B* were 6.24 and 4.79 cM, respectively. The location of *TaARF4-B* on chromosome 3B was validated using diploid wild relative species and nulli-tetrasomic lines of Chinese Spring (Supplementary Data Fig. S3B).

### Association analysis of *TaARF4-B* haplotypes and agronomic traits

**Fig. 8.**
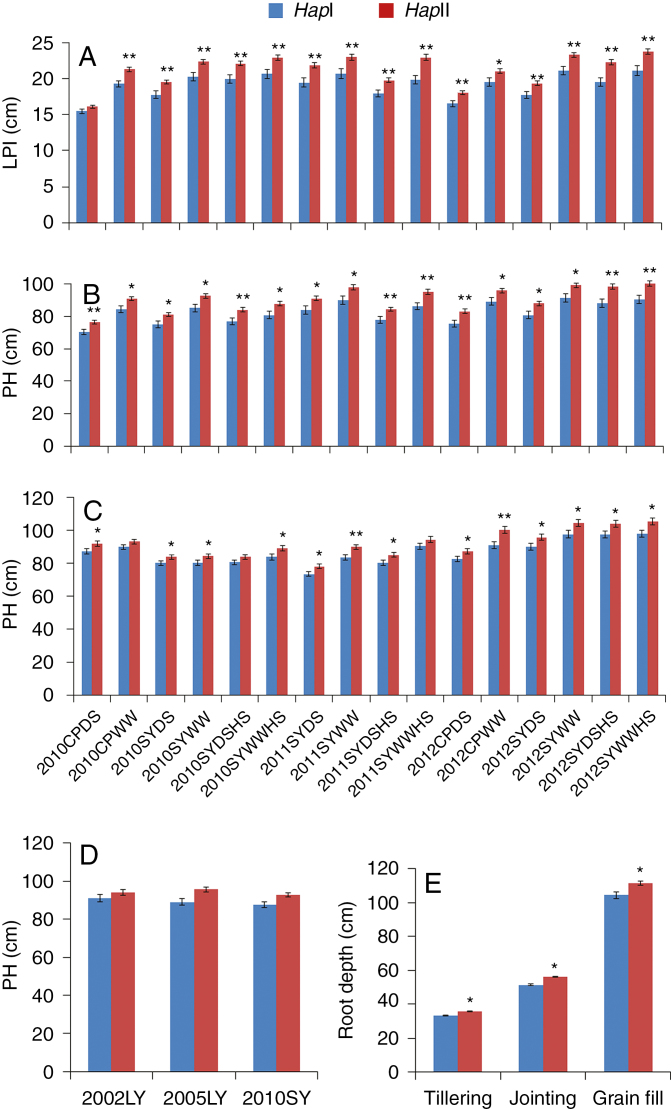
Phenotypic comparisons of two *TaARF4-B* haplotypes. (A) LPI (length of penultimate internode) comparisons of two *TaARF4-B* haplotypes in Population 2 in 16 environments. (B) PH (plant height) comparisons of two *TaARF4-B* haplotypes in Population 2 in 16 environments. (C) PH comparisons of two *TaARF4-B* haplotypes in Population 1 in 16 environments. (D) PH comparisons of two *TaARF4-B* haplotypes in Population 5 in three environments. (E) Root depth comparisons of two *TaARF4-B* haplotypes in Population 3 at tillering, jointing and grain fill stages. Error bars are 2 × s.e. *P < 0.05, **P < 0.01 (Student’s *t*-test).

To investigate the phenotypic effects of the two *TaARF4-B* haplotypes, Population 2 consisting of 262 accessions was used for association analysis of a range of agronomic traits. Among 16 environments (years × sites × water regimes × heat treatment), significant associations between *TaARF4-B* and LPI were identified in 14 environments (except 2010CPDS and 2012CPWW). *TaARF4-B* was strongly associated with PH in 14 environments (except 2011SYWW and 2012SYWW) (Table 1). The phenotypic variation in LPI explained by *TaARF4-B* ranged from 2.44 to 10.54 %, and the phenotypic variation in PH explained by *TaARF4-B* ranged from 4.26 to 8.82 %. Accessions with *Hap*I had significantly shorter LPI than those with *Hap*II in all the environments except 2010CPDS. *Hap*I reduced LPI by 1.47–2.75 cm compared with *Hap*II (Fig. 8A). Accessions possessing *Hap*I had significantly shorter PH than those with *Hap*II in all 16 environments. *Hap*I reduced PH by 5.97–10.35 cm compared with *Hap*II (Fig. 8B).

**Table 1.** *TaARF4-B* haplotypes associated with agronomic traits in 16 environments

Year	Site	Environment	LPI		PH	
			*P*-value	*PVE* (%)	*P*-value	*PVE* (%)
2010	CP	DS	n.s.	2.44	0.0247*	7.16
	CP	WW	0.0022***	6.00	0.0374*	5.26
	SY	DS	0.0185*	7.42	0.0472*	6.27
	SY	WW	0.0105*	5.42	0.0288*	4.64
	SY	DS–HS	0.0184*	7.55	0.0401*	5.53
	SY	WW–HS	0.0115*	6.76	0.0361*	6.52
2011	SY	DS	0.0327*	8.32	0.0498*	6.21
	SY	WW	0.0161*	7.28	n.s.	5.34
	SY	DS–HS	0.0148*	8.31	0.0305*	5.89
	SY	WW–HS	5.79E-04***	7.75	0.0130*	4.88
2012	CP	DS	0.0129*	6.21	0.0141*	7.20
	CP	WW	n.s.	3.85	0.0406*	4.88
	SY	DS	0.0204*	3.59	0.0422*	4.26
	SY	WW	0.0126*	5.82	n.s.	4.75
	SY	DS–HS	0.0075**	10.19	0.0107*	8.12
	SY	WW–HS	0.0046***	10.54	0.0143*	8.82

LPI, length of penultimate internode; PH, plant height; n.s., not significant; *P < 0.05, **P < 0.01 and ***P < 0.001; *PVE*, phenotypic variation explained. The environments were at Changping (CP) and Shunyi (SY) under well-watered (WW), drought-stressed (DS) and/or heat-stress (HS) conditions in 2010–2012.

The effects of *TaARF4-B* haplotypes were confirmed in two other populations. Accessions possessing *TaARF4-B Hap*I in Population 1 also had significantly shorter PH than those with *Hap*II in 13 of the 16 environments. *Hap*I reduced PH by 3.67–9.10 cm compared with *Hap*II (Fig. 8C). Similar results were obtained for Population 5, where *Hap*I reduced PH from 5.23 to 6.67 cm relative to *Hap*II (Fig. 8D). The results suggested that the *TaARF4* haplotype affected plant growth, and *TaARF4 Hap*I was considered a superior allele for reducing PH.

### Association analysis between *TaARF4-B* haplotypes and root phenotype

We also performed an association analysis between *TaARF4-B* haplotypes and root depth phenotype at the seedling, tillering, jointing and grain fill stages. Significant associations were identified between haplotypes and root depth at tillering (*P* = 0.035, *PVE* = 4.50 %), jointing (*P* = 0.020, *PVE* = 5.48 %) and grain fill (*P* = 0.015, *PVE* = 5.94 %) stages. *Hap*I accessions had shallow roots, which were 2.46 cm shorter compared with those of *Hap*II at tillering, 4.71 cm at jointing and 7.00 cm at grain fill stages (Fig. 8E).

### Geographic distribution of *TaARF4-B* haplotypes in ten Chinese wheat zones

**Fig. 9.**
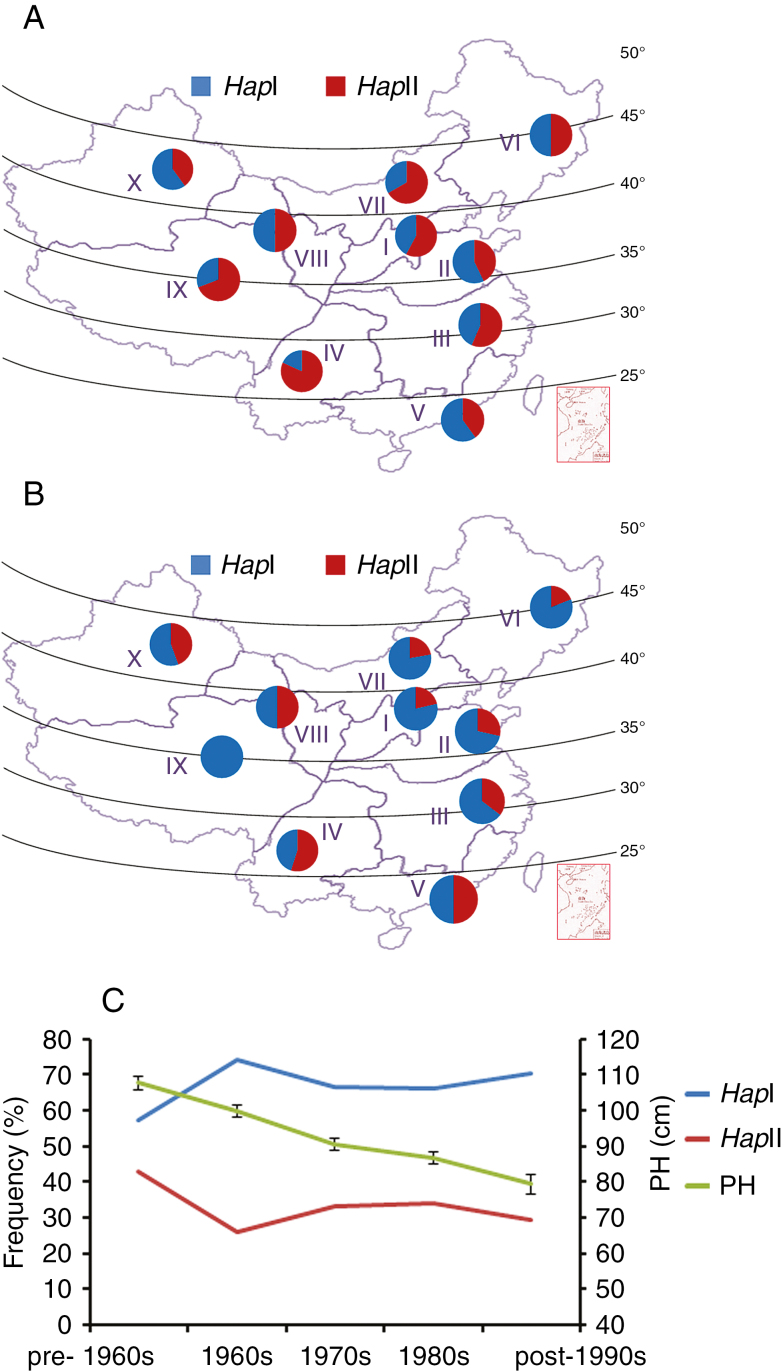
Haplotype distribution and frequency change of *TaARF4-B*. (A) Distribution of two *TaARF4-B* haplotypes in 157 landraces of ten Chinese major wheat zones. (B) Distribution of two *TaARF4-B* haplotypes in 348 modern cultivars of ten Chinese major wheat zones. I, Northern Winter Wheat Region; II, Yellow and Huai River Valley Winter Wheat Region; III, Low and Middle Yangtze River Valley Winter Wheat Region; IV, South-western Winter Wheat Region; V, Southern Winter Wheat Region; VI, North-eastern Spring Wheat Region; VII, Northern Spring Wheat Region; VIII, North-western Spring Wheat Region; IX, Qinghai–Tibet Spring–Winter Wheat Region; X, Xinjiang Winter–Spring Wheat Region. (C) Haplotype frequency change of *TaARF4-B* in Chinese wheat breeding programmes. The left *y*-axis shows frequencies of *TaARF4-B* in Population 5 over decades. The right *y*-axis shows PH in Population 5 over decades. Accessions released in the pre-1960s, 1960s, 1970s, 1980s and post-1990s are numbered 37, 55, 102, 106 and 34, respectively. Thirteen accessions with unknown release dates were excluded. Error bars are 2 × s.e.

Chinese wheat production regions are divided into ten ecological zones based on growing season and ecological conditions. Landraces (Population 4) and modern cultivars (Population 5) were used to investigate the distribution of *TaARF4-B* haplotypes (Fig. 9A, B). For the landraces, *Hap*II was the dominant haplotype in seven zones [except Zone II (43 %), Zone V (40 %) and Zone X (40 %)]. The average frequency of *Hap*II was 56 %. For modern cultivars, *Hap*I was the dominant haplotype in almost all zones, with the only exception being Zone IV (45 %). The average frequency of *Hap*I was 67 %. From Chinese landraces to modern cultivars, the frequencies of *Hap*I increased across eight zones [with the exception of Zone V (from 60 to 50 %) and Zone X (from 44 to 40 %)]. Comparisons of landraces and modern cultivars indicated that *TaARF4-B Hap*I was positively selected in wheat breeding.

### *TaARF4-B* haplotypes were selected during wheat breeding

Wheat breeding is a process of accumulating favourable haplotypes, and leaves footprints in genomes. In order to verify further that selection had occurred for a particular haplotype, we determined haplotype frequency changes according to cultivar release dates. PH continually declined from pre-1960s to post-1990s, whereas the frequency of *Hap*I increased from 57.1 % pre-1960s to 74.1 % in the 1960s (Fig. 9C). After the 1960s, the frequency of *Hap*I remained stable while PH continued to decline, implying selection of reduced height based on other genes.

## DISCUSSION

Optimizing root and plant architecture is regarded as an important objective for wheat breeding. Therefore, it is essential to understand the molecular mechanisms of root growth and plant development. Here, we identified a novel ARF gene family member, *TaARF4*, and demonstrated that *TaARF4* overexpression in arabidopsis caused shorter primary root length and PH. We propose that this effect occurs by repressing *GH3* gene expression to mediate IAA homeostasis. Meanwhile, association analysis results showed that variation in *TaARF4-B* was significantly associated with root depth and PH. Therefore, *TaARF4* may be an important gene resource for regulating wheat growth, and dCAPS markers of *TaARF4* might be useful for the selection of wheat genotypes with optimal plant architecture.

### ARFs and their target genes

ARFs as transcription activators or repressors participate in the auxin pathway, and affect auxin-responsive gene expression (Hagen and Guilfoyle, 2002; Weijers *et al.*, 2018). Identification of the target genes for ARFs is a major step in studying their function. Previous research by ChIP, EMSA and gene expression analyses proved that *Aux*/*IAA* genes are direct targets of ARF5/MP transcriptional regulation (Krogan *et al.*, 2014). *ATHB8* (*Arabidopsis thaliana Homeobox Gene 8*) is a target of ARF5/MP which regulates pre-procambial cell state acquisition by auxin signalling in arabidopsis leaves (Donner *et al.*, 2009). *DRN* (*DORNROSCHEN*) is another target of ARF5/MP in the arabidopsis embryo (Cole *et al.*, 2009). *PLT* (*PLETHORA*) transcription requires ARF5/MP and NPH4/ARF7 to mediate embryonic root patterning (Aida *et al.*, 2004). In rice, *CRL1* (*crown rootless 1*) is a target of ARF in crown root formation (Inukai *et al.*, 2005). Thus, ARFs target different genes and have different functions. Our research indicates that overexpression of *TaARF4* leads to ABA insensitivity by targeting *HB33*, and reduces root length and PH by targeting *GH3* genes.

### TaARF4, GH3s and IAA homeostasis

Indole-3-acetic acid, as the major auxin form in plants, plays an important role in plant growth (Benková *et al.*, 2003; Mockaitis and Estelle, 2008). Biosynthesis, catabolism and conjugation of IAA significantly affect plant development (Ljun *et al.*, 2002; González-Lamothe *et al.*, 2012). IAA has many conjugate forms, such as ester conjugates with sugars, and amide conjugates with amino acids (Pencik *et al.*, 2009). IAA amide forms IAA-Ala, IAA-Leu and IAA-Phe are forms that store IAA, whereas IAA-Asp and IAA-Glu are degradation precursors (Ludwig-Müller, 2011). These forms maintain IAA homeostasis. GH3s have roles in multiple processes (Ludwig-Müller *et al.*, 2009). There are 19 GH3 family members in arabidopsis, and at least seven members (GH3.2–GH3.6, GH3.9 and GH3.17) are encoded by group II genes and can catalyse IAA amide synthesis (Staswick *et al.*, 2005). In this study, we showed that *TaARF4* overexpression in arabidopsis led to low *GH3* gene expression and higher free IAA content. The high free IAA that inhibits apical dominance also reduced primary root length and PH.

### *TaARF4-B* haplotypes and phosphorylation status

Thirteen variants were identified among two haplotypes of *TaARF4-B*, but only one amino acid change was discovered, threonine to alanine at amino acid position 158. This amino acid is located in a conserved DBD. Threonine can be phosphorylated whereas alanine is a dephosphorylated site. Using the NetPhos 3.1 Server, we predicted phosphorylated sites in TaARF4 and found that this site can be phosphorylated by DNA-dependent protein kinase (Supplementary Data Fig. S4). This predicts that *TaARF4-B Hap*I might be phosphorylated at amino acid position 158, whereas *Hap*II cannot be phosphorylated at this site. In plants, phosphorylation of transcription factors is an important regulatory mechanism, especially in hormone signalling pathways (Hill, 2015). For example in arabidopsis, the phosphorylation status of BZR1/2 affects target gene expression in the brassinosteroid (BR) signalling pathway (Wang *et al.*, 2012). In the ABA signalling pathway, the phosphorylation status of RAV1 affects expression of *ABI3*, *ABI4* and *ABI5* (Feng *et al.*, 2014). Similarly, the phosphorylation status of ARF2 is important to ARF2 function. In the BR signalling pathway, ARF2 can be phosphorylated by BIN2, resulting in loss of DNA binding and repression activities (Vert *et al.*, 2008). Low potassium triggers a Ser689 phosphorylation in ARF2 and relieves its repression on *HAK5* (Zhao *et al.*, 2016). So different *TaARF4-B* haplotypes in wheat accessions may lead to a difference in TaARF4-B phosphorylation status, which in turn may lead to different root depth and PH.

## SUPPLEMENTARY DATA

Supplementary data are available online at https://academic.oup.com/aob and consist of the following. Figure S1: structural analysis and phylogenic tree of TaARF4 protein and homologous ARF proteins. Figure S2: semi-quantitative PCR detecting *TaARF4-A* expression levels in arabidopsis. Figure S3: mapping of *TaARF4-B* on wheat chromosomes. Figure S4. potential phosphorylation sites in TaARF4-B predicted by the NetPhos 3.1 Server. Table S1: primers used for real-time PCR. Table S2: primers used for vector construction. Table S3: primers used for ChIP assays. Table S4: primers used for genomic fragment isolation, sequencing and marker development. Table S5: accession names in Populations 2, 3, 4 and 5.

## Supplementary Material

mcy218_suppl_Supplementary_Material
