# Supplementary material for: TaARF4 genes are linked to root growth and plant height in wheat
Source: Ann Bot. 2018 Dec 24;124(6):903–15. doi: 10.1093/aob/mcy218 (PMC6881231; doi:10.1093/aob/mcy218)
Supplement: mcy218_suppl_Supplementary_Material [file mcy218_suppl_supplementary_material.docx]

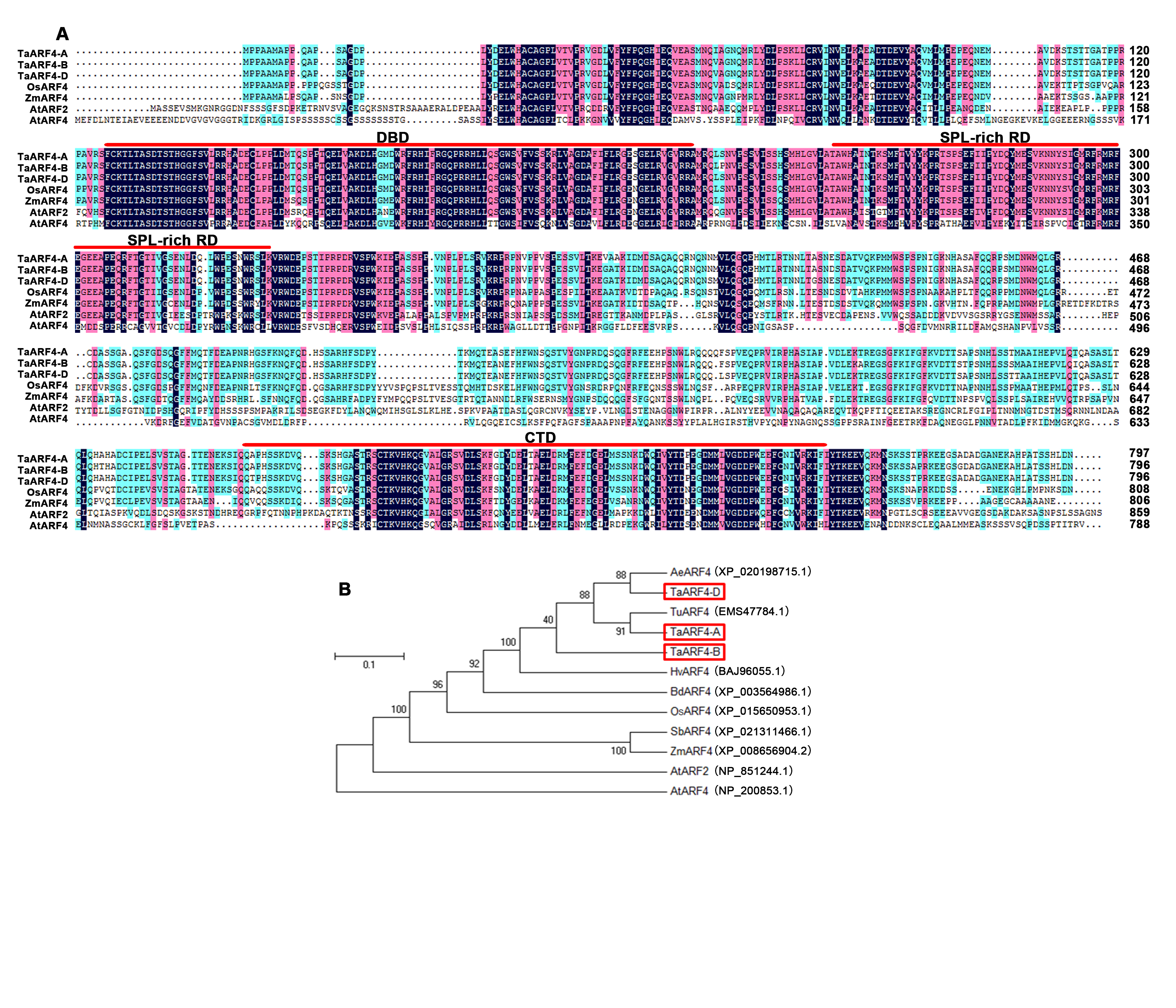


Figure S1. Structural analysis and phylogenic tree of TaARF4 protein and homologous ARF proteins.

(A) Amino acid sequences alignment of TaARF4 and homologous proteins. ARFs are comprised of DBD (DNA binding domain), SPL-rich RD (repression domain) and a CTD (C-terminal domain), that are marked by lines above the sequences. Amino acids shaded by color are conserved, black shaded amino acids indicate the highest similarity, red less, and blue least.

(B) Phylogenic tree of TaARF4 and homologous ARF proteins. Ae, *Aegilops tauschii*; At, *Arabidopsis* *thaliana*; Bd, *Brachypodium distachyon*; Hv, *Hordeum vulgare*; Os, *Oryza sativa*; Zm, *Zea mays*; Sb, *Sorghum bicolor*; Ta, *Triticum aestivum*; Tu, *Triticum urartu*.


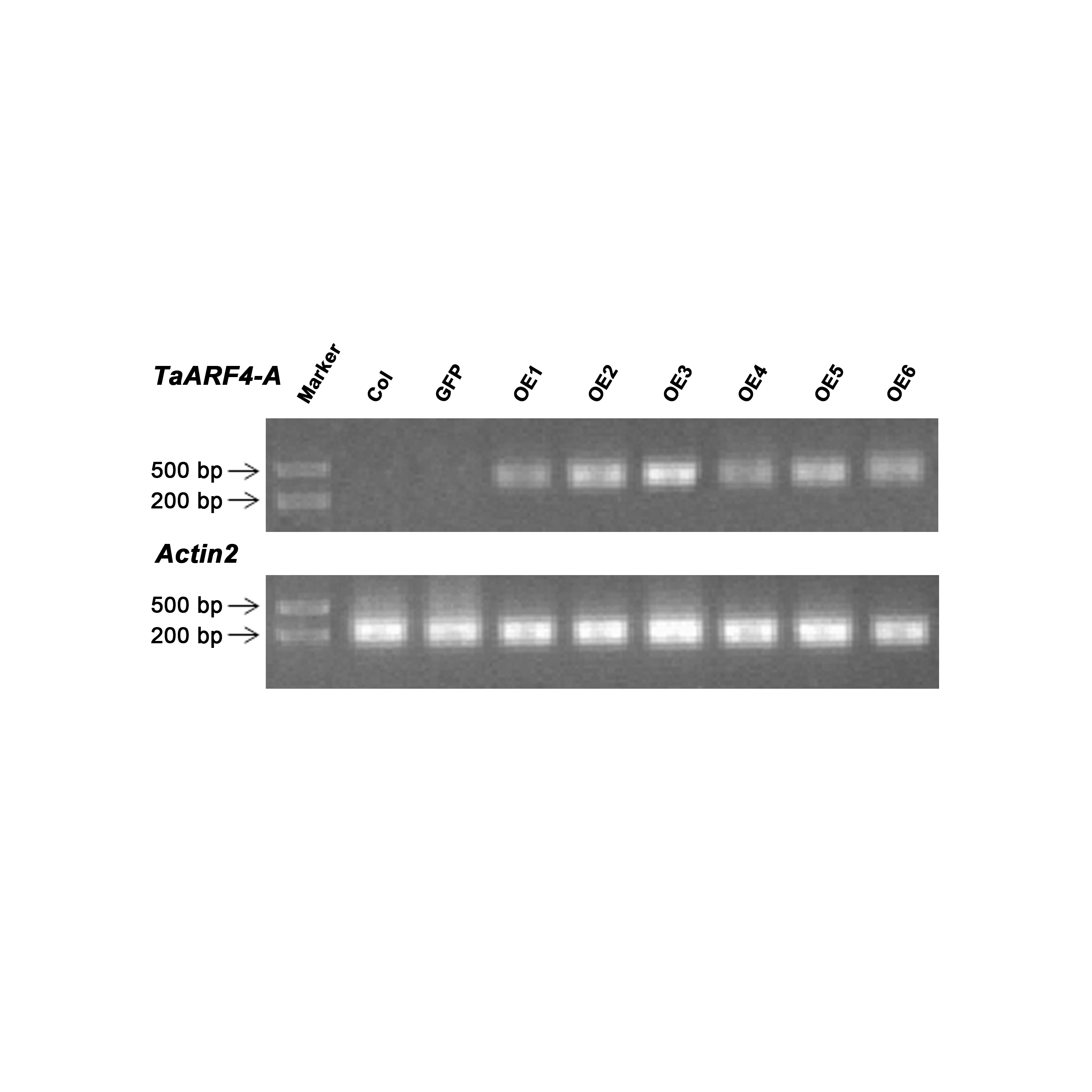


Figure S2. Semi-quantitative PCR detecting *TaARF4-A* expression levels in *Arabidopsis*.


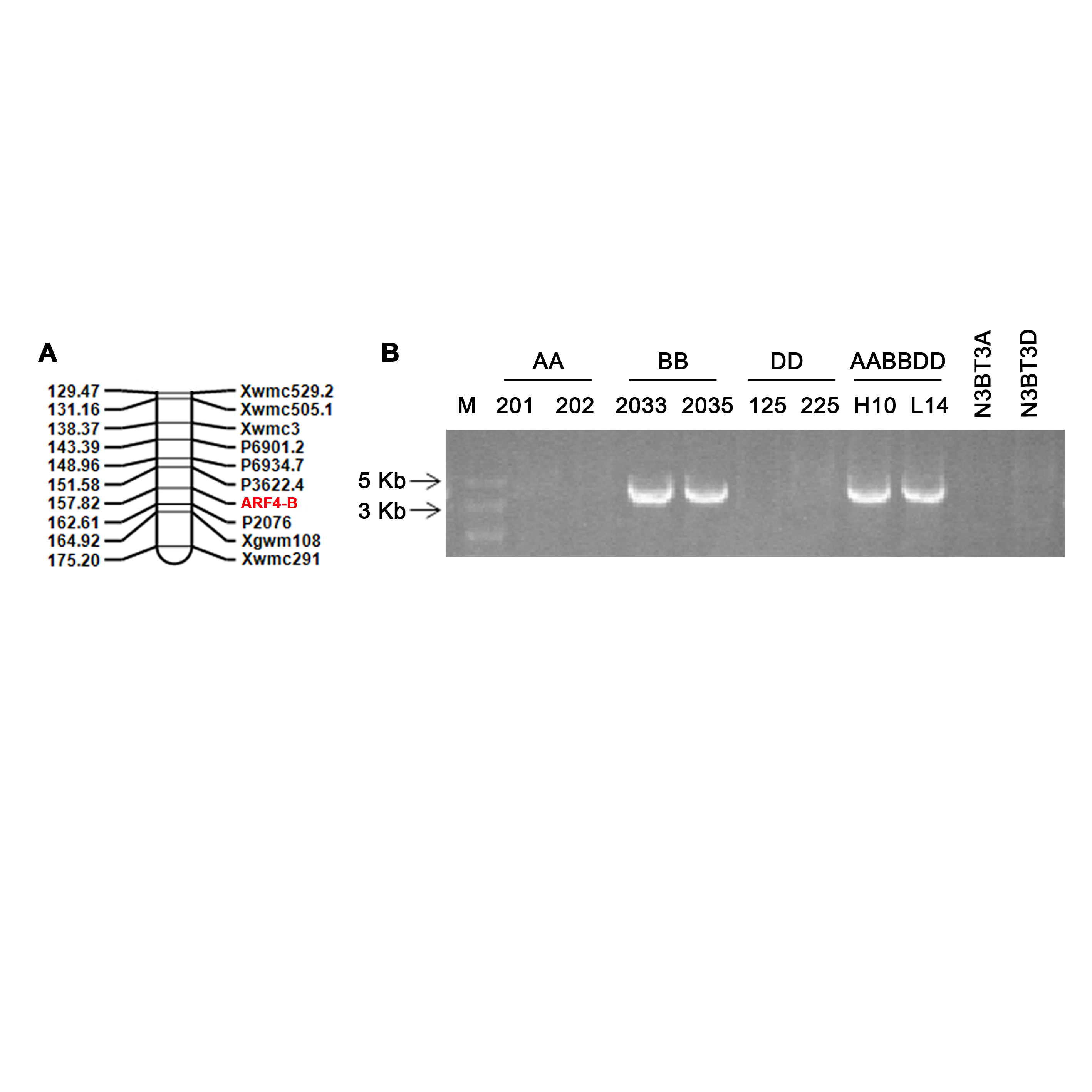


Figure S3. Mapping of *TaARF4-B* on wheat chromosomes.

(A) *TaARF4-B* was mapped on chromosome 3B flanked by *P3622.4* and *P2076*.

(B) *TaARF4-B* was located on chromosome 3B using diploid accessions (AA, 201 and 202; BB, 2033 and 2035; DD, 125 and 225), hexaploid accessions (AABBDD, Hanxuan 10 and Lumai 14) and nulli-tetrasomic lines of Chinese Spring (N3BT3A and N3BT3D).


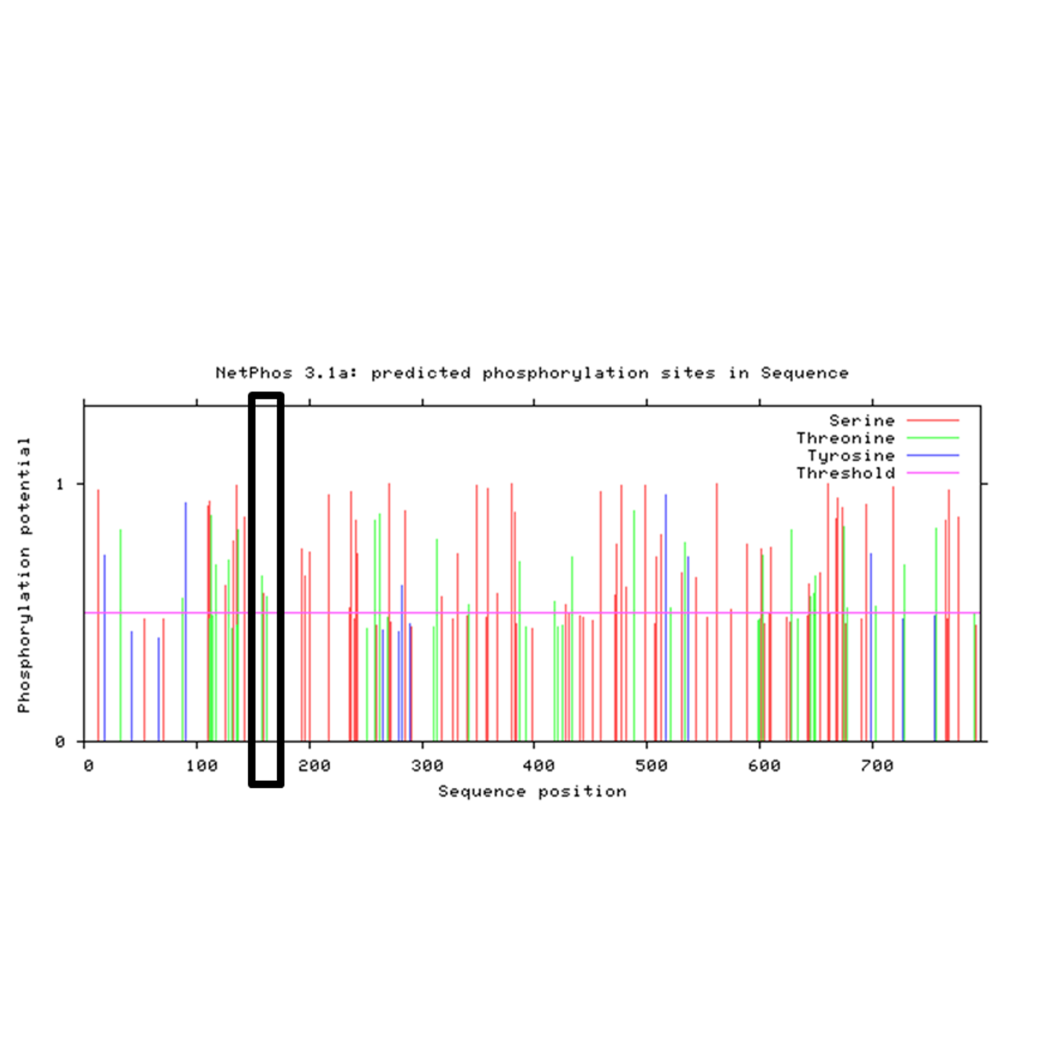


Figure S4. Potential phosphorylation sites in TaARF4-B predicted by the NetPhos 3.1 Server.

Black box showed amino acid change at amino acid position 158 between different haplotypes.

Table S1. Primers used for real-time PCR

| **Primer name** | **Primer sequence (5' to 3')** |
| --- | --- |
| RT-TaARF4-F1 | TCATTTCTGGAATAGCCAAAGCACCG |
| RT-TaARF4-R1 | CAGCTCAGGAATACAATCAGCGTGTG |
| RT-GAPDH- F | CTGCATCATACGATGACATC |
| RT-GAPDH- R | TGTCACCGACAAAGTCAGTG |
| RT-HB33-F | GGACAATCAAGCGGAGAAGGAGC |
| RT-HB33-R | CTCCGATCTCGCCGCAGAATCTC |
| RT-GH3.2-F | GAAGAAGTCATGGCTAAGTGCTGTTTGGAG |
| RT-GH3.2-R | GTCAAGCAGCTCCATGATAGGTGTGAAG |
| RT- GH3.5-F | GGAAGGGTTAGTGACAAGTCCATAGGC |
| RT- GH3.5-R | GTTACTCCCCCACTGTTTGTGACC |
| RT-Tubulin-F | GAGGCCTCGTGTGGTCGCTTTGT |
| RT-Tubulin-R | GCCCAGTTGTTACCCGCACCAGA |

Table S2. Primers used for vector construction

| **Primer name** | **Primer sequence (5' to 3')** | **Vector** | | **Restriction enzyme** |
| --- | --- | --- | --- | --- |
| cTaARF4-A-F1 | CTAGGAATTCATGGCGCCGCCGCAGGCC | pGEX-4T1 | *Eco* RI | |
| cTaARF4-A-R1 | TGACCTCGAGAATCGGCTTAACGCAACCTTCCTC |  | *Xho* I | |
| cTaARF4-A-F1 | CTAGGAATTCATGGCGCCGCCGCAGGCC | pGEX-4T1 | *Eco* RI | |
| cTaARF4-A-R2 | TGACCTCGAGCGGAATAGTTGACGGCTCATCCCAAC |  | *Xho* I | |
| cTaARF4-A-F3 | CTAGTCTAGAATGGCGCCGCCGCAGGCC | pCAMBIA1300 | *Xba* I | |
| cTaARF4-A-R3 | TGACACTAGTGAATCGGCTTAACGCAACCTTCCTC |  | *Spe* I | |

Table S3. Primers used for ChIP assays

| **Primer name** | **Primer sequence (5' to 3')** |
| --- | --- |
| CHIP-HB33-F1 | CATCTCTCACAAAGGGCTCAGC |
| CHIP-HB33-R1 | GCTCAAAGCTTTTTGCTTGTGAAGG |
| CHIP-HB33-F2 | CACCACCACAAGTATGGAATGAGTCC |
| CHIP-HB33-R2 | GTCCATCATCCTCTCCTTCTGATCC |
| ChIP-GH3.2-F1 | CCAGAACGACGCCTATGAAACCAATAAGAC |
| ChIP-GH3.2-R1 | CAATGTCCACGTGGACGAGAGTACTTTAG |
| ChIP-GH3.2-F2 | GGAACATCTGCTGGTGAGAGGAAAC |
| ChIP-GH3.2-R2 | CGTCTTTGACTCCGACTTCACGAATAAG |
| ChIP- GH3.5-F1 | CGGAAAGAGAGAAAAAGAGTCGCAATCC |
| ChIP- GH3.5-R1 | GTGTGTGAGGAAGAAAGAAAGAGAAAGGGTC |
| ChIP- GH3.5-F2 | Cttctgggtacccaaaaggttgaaac |
| ChIP- GH3.5-R2 | GAAATACATTCCTTTGCCGTTTTCGAGAC |
| ChIP-Tubulin-F | TATGGTCAAGGCTGGGTTCG |
| ChIP-Tubulin-R | CCATGCTCGATGGGGTACTT |

Table S4. Primers used for genomic fragment isolation, sequencing and marker development

| **Primer name** | **Primer sequence (5' to 3')** | **Experimental purpose** |
| --- | --- | --- |
| TaARF4-A-F1 | ACCACAGTCACAGTCGGCTC | A genome-specific primers for SNP detection |
| TaARF4-A-R1 | GTGATGCGAATCATATCATGGCGCAAAC |  |
| TaARF4-B-F1 | CTCCGCTACTACTACTCCTCTCTCCTC | B genome-specific primers for SNP detection |
| TaARF4-B-R1 | CAAATCGGCTTAACGCAACCTGCAG |  |
| TaARF4-D-F1 | CCGCCCTCCCCACAAAACATTTC | D genome-specific primers for SNP detection |
| TaARF4-D-R1 | CATCCGCAGAACCTTCCTCCTTTCTC |  |
| TaARF4-ABD-CF1 | GGGAAATTCGATTCGGTCAGCTGATTTG | DNA sequence primers |
| TaARF4-ABD-CF2 | CATCGATGATAATTGTGTTAACCGAGTG |  |
| TaARF4-ABD-CF3 | GTCATGTAGGTTTACTGGTACTATAGTTGGCAGTG |  |
| TaARF4-ABD-CF4 | CTTCTTCATGCAGACCTTTGATGAGGCTC |  |
| TaARF4-A-CF | GTCAGTTCCAAAAGGCTTGTAGCTGG | cDNA sequence primers |
| TaARF4-A-CR | GATGCATGAGGCCGGATCACTC |  |
| dCAP-TaARF-B-F | GAAGTAAGCTCTACCGTGAATTTCGCTGG | dCAPS primers (*Sac* I) |
| TaARF-B-SacI-R | CGTCAGAGTCTTGCAGAAGGAGCT |  |

Table S5. Accession names in Population 2, 3, 4 and 5

| **Population2** | | **Population3** | | **Population4** | | **Population5** | |
| --- | --- | --- | --- | --- | --- | --- | --- |
| No. | Name | No. | Name | No. | Name | No. | Name |
| 1 | Drysdale | 1 | Drysdale | 1 | Chanbuzhi | 1 | Anhui 3 |
| 2 | Rees | 2 | Salgemma | 2 | Dahuangpi | 2 | Anhui 9 |
| 3 | Wanmai 19 | 3 | Bainong 160 | 3 | Jiangxizao | 3 | Yimai 1 |
| 4 | An85 | 4 | Bo'ai 7023 | 4 | Xianmai | 4 | Huadong 10 |
| 5 | An86 | 5 | Dali 1 | 5 | Chongyanghongmai 1 | 5 | Huadong 6 |
| 6 | Beijing 10 | 6 | Dali 52 | 6 | Honghuazao | 6 | Liying 1 |
| 7 | Beijing 14 | 7 | Fanmai 8 | 7 | Sankecun | 7 | Liying 5 |
| 8 | Beijing 837 | 8 | Fengchan 1 | 8 | Heshangmai | 8 | Wangmai 17 |
| 9 | Beijing 8686 | 9 | Fengchan 3 | 9 | Nuomai | 9 | Jiulan |
| 10 | Beijing 8694 | 10 | Fengkang 13 | 10 | Chejianzi | 10 | Anhui 11 |
| 11 | Beinong 2 | 11 | Fuzhuang 30 | 11 | Jiangdongmen | 11 | Mengfeng 8 |
| 12 | Dan R8043 | 12 | Han05-5092 | 12 | Liuzhutou | 12 | Emai 6 |
| 13 | Dan R8093 | 13 | Han 6172 | 13 | Mangxiaomai | 13 | Jingzhou 2 |
| 14 | Dan R8108 | 14 | Handan 6050 | 14 | Wangshuibai | 14 | Zhongjiwan |
| 15 | Dan R8194 | 15 | Heng 216 | 15 | Zaowutian | 15 | Zhongshan 9 |
| 16 | Dan R9062 | 16 | Heng 4399 | 16 | Paozimai | 16 | Wanyuan 28 |
| 17 | Dongxie 2 | 17 | Heng 5229 | 17 | Shuilizhan | 17 | Xiangmai 8 |
| 18 | Fengkang 13 | 18 | Heng 7228 | 18 | Wuyuanmai | 18 | Xiang 791-2 |
| 19 | Fengyou 5 | 19 | Heng 95 guan 26 | 19 | Baipu | 19 | Zhongana 875 |
| 20 | Huabei 187 | 20 | Hengguan 35 | 20 | Huangshuibai | 20 | Nannong 2293xuan |
| 21 | Jian 26 | 21 | Hengmai 2 | 21 | Lanxizaoxiaomai | 21 | Siyang 117 |
| 22 | Jing 411 | 22 | Hengshui 6404 | 22 | Zaoxiaomai | 22 | Sujian 14 |
| 23 | Jingdong82 | 23 | Hengyou 18 | 23 | Zhumaoyuanzitou | 23 | Sumai 3 |
| 24 | Jingdong83 | 24 | Huaimai 18 | 24 | Baiyoumai | 24 | Jiamai 25 |
| 25 | Jingdong 8 | 25 | Huaimai 25 | 25 | Daqingmang | 25 | Nanzhongzao |
| 26 | Jinghe 8922 | 26 | Huaishu 10 | 26 | Guangtou | 26 | Zhemai 1 |
| 27 | Jinghua 1 | 27 | Lantian 15 | 27 | Yangmai | 27 | Zhexuan 78-23 |
| 28 | Jingnong 79-15 | 28 | Liangxing 99 | 28 | Huoqiu | 28 | Wan 85-50-Fan3 |
| 29 | Jingnong80 | 29 | Lovrin 10 | 29 | Donghuachunmai | 29 | Wanpin 8203 |
| 30 | Jingnong 84-6789 | 30 | Luohan 11 | 30 | Dabaipi | 30 | Wanpin 8337 |
| 31 | Jingpin 11 | 31 | Luohan 13 | 31 | Hongpixiaomai | 31 | E 811 |
| 32 | Jingpin 30 | 32 | Luohan 2 | 32 | Huoliaomai | 32 | Enmai 4 |
| 33 | Jingpin 3 | 33 | Luohan 3 | 33 | Xiaobaimai | 33 | Exi 84-1031 |
| 34 | Jingshuang 16 | 34 | Luohan 6 | 34 | Xiaohongpi | 34 | Xiangmai 12 |
| 35 | Jingshuang 2 | 35 | Luohan 7 | 35 | Chunxiaomai | 35 | Kang R16 |
| 36 | Jingxuan 20 | 36 | Luohan 8 | 36 | Honglidangnianlao | 36 | Nanda 8910 |
| 37 | Jingxuan 25 | 37 | Luohan 9 | 37 | Dahongmai | 37 | Ning 8343 |
| 38 | Jingyan85 jian28 | 38 | Luomai 21 | 38 | Dingxingzhai | 38 | Ning 8537 |
| 39 | Keyi 26 | 39 | Luomai 23 | 39 | Baiqimai | 39 | Ning 8924 |
| 40 | Keyi 29 | 40 | Luonong 10 | 40 | Hongjinmai | 40 | Ningmaizi 13 |
| 41 | Lunkang 7 | 41 | Luoyang 8628 | 41 | Lanhuamai | 41 | Ningmaizi 19 |
| 42 | Lunxuan 987 | 42 | Luomai 8 | 42 | Shanxibaimai | 42 | Ningmaizi 3 |
| 43 | Nongda 135 | 43 | Luomai 9 | 43 | Baiqiumai | 43 | Limai 16 |
| 44 | Nongda 146 | 44 | Luoyou 7 | 44 | Hongmai | 44 | Zhemai 4 |
| 45 | Nongda 155 | 45 | Qingchun 1 | 45 | Xiaobaimang | 45 | Wan 89193 |
| 46 | Nongda 183 | 46 | Qingchun 2 | 46 | Zhuoludongmai | 46 | Wanpin 8410 |
| 47 | Nongda 20074 | 47 | Qingshan 843 | 47 | Mahuaban | 47 | Ningmaizi 44 |
| 48 | Nongda 311 | 48 | Shi 4185 | 48 | Panshiwumang | 48 | Yangmai 158 |
| 49 | Nongda 33 | 49 | Shijiazhuang 407 | 49 | Youmangbaifu | 49 | Exi 652 |
| 50 | Nongda 36 | 50 | Shijiazhuang 8 | 50 | Xiaokouhong | 50 | Yixi 102 |
| 51 | Nongda 81146 | 51 | Shimai 12 | 51 | Youmangbaifu | 51 | Gan 162 |
| 52 | Xiaoshan 8 | 52 | Shimai 13 | 52 | Honglaomai | 52 | Dongnong 101 |
| 53 | Xiaoyan 54 | 53 | Shimai 15 | 53 | Laomai | 53 | Hezuo 3 |
| 54 | Xiaoyan 81 | 54 | Shimai 18 | 54 | Hongpidongmai | 54 | Jia 6268A-549 |
| 55 | Yanda 1817 | 55 | Shimai 19 | 55 | Jiahongmai | 55 | Kequn |
| 56 | Yuandong 3 | 56 | Xuzhou 21 | 56 | Niuzhijia | 56 | Xinshuguang 1 |
| 57 | Yuandong 834 | 57 | Xuzhou 6 | 57 | Daimanghongmai | 57 | Xinshuguang 6 |
| 58 | Yuandong 847 | 58 | Yanzhan 1 | 58 | Baidatou | 58 | Hei 78-1259 |
| 59 | Yuandong 856 | 59 | Yubao 1 | 59 | Baiqitou | 59 | Kefeng 3 |
| 60 | Yuansheng 215 | 60 | Yumai 13 | 60 | Dabaimai | 60 | Kelao 4 |
| 61 | Zaosui 21 | 61 | Yumai 18 | 61 | Hongmangmai | 61 | Kenbei 1 |
| 62 | Zaosui 65 | 62 | Yumai 29 | 62 | Huangjinmai | 62 | Kenda 1 |
| 63 | Zaosui 66 | 63 | Yumai 2 | 63 | Huoliyan | 63 | Long 79-9468 |
| 64 | Zhong 7902 | 64 | Yumai 38 | 64 | Yizhimai | 64 | Fengqiang 3 |
| 65 | Zhong 8502 | 65 | Yumai 47 | 65 | Baimazha | 65 | Heifu 84S1378 |
| 66 | Zhongda 86 | 66 | Yumai 48 | 66 | Hongtuzi | 66 | Jia 84-S437 |
| 67 | Zhongda 91 | 67 | Yumai 8 | 67 | Laotutou | 67 | Longfumai 2 |
| 68 | Zhongda 92 - jian 49 | 68 | Yunong 416 | 68 | Shanmai | 68 | Longmai 18 |
| 69 | Zhongda 92 - pin 8 | 69 | Yunong 949 | 69 | Shanmai | 69 | Longmai 19 |
| 70 | Zhonghan 110 | 70 | Yuzhan 4 | 70 | Dabaimai | 70 | Xinkehan 9 |
| 71 | Zhongmai 9 | 71 | Zhoumai 16 | 71 | Galaohan | 71 | Jichun 1016 |
| 72 | Zhongsu 68 | 72 | Zhoumai 18 | 72 | Gejiaxiang | 72 | Kenhong 15 |
| 73 | Zhongyou 9507 | 73 | Zhoumai 22 | 73 | Liuyuehuang | 73 | Xiaobingmai 33 |
| 74 | Zhongzuo 60064 | 74 | Zhoumai 23 | 74 | Baimangxiaomai | 74 | Bihongsui |
| 75 | Zhongzuo 60115 | 75 | Shite 14 | 75 | Bendihuanghuamai | 75 | Lianglaiyoubaipixiaomai |
| 76 | Hongliang 4 | 76 | Shiyou 17 | 76 | Dachunbaisilengmai | 76 | Jinghong 5 |
| 77 | H 89 | 77 | Shiyou 20 | 77 | Kangdingxiaomai | 77 | Jiba 7529 |
| 78 | Jin 2148 - 7 | 78 | Wanmai 19 | 78 | Bailanghuimai | 78 | Neimai 11 |
| 79 | Baiqimai | 79 | Wenmai 6 | 79 | Bianbachunmai 6 | 79 | Jinchun 3 |
| 80 | Longjian 196 | 80 | Xi'an 8 | 80 | Geerhongmai | 80 | Yanbei 8 |
| 81 | Longjian 294 | 81 | Xinong 1018 | 81 | Motuoxiaomai | 81 | Pinchun 14 |
| 82 | Pingliang 35 | 82 | Xinong 189 | 82 | Muzongzhuoga | 82 | Jichun 8055-1 |
| 83 | Qingshan 843 | 83 | Xinong 219 | 83 | Wujiangzhuo | 83 | Dingxian 72 |
| 84 | Qingfeng 1 | 84 | Xinong 318 | 84 | Zhahong | 84 | Huabei 672 |
| 85 | Xifeng 16 | 85 | Xinong 6028 | 85 | Shengen | 85 | Nongda 183 |
| 86 | Xifeng 20 | 86 | Xinong 688 | 86 | Songruimai | 86 | Henong 3 |
| 87 | Xifeng 9 | 87 | Xinong 928 | 87 | Chaoanxiaomai | 87 | Mingxian 169 |
| 88 | Zhangdong 29 | 88 | Xinong 9106 | 88 | Chike | 88 | Beijing 8 |
| 89 | Bawangbian | 89 | Xinmai 296 | 89 | Shanglinxiaomai | 89 | Dongfanghong 3 |
| 90 | Cangmai 6001 | 90 | Jimai 19 | 90 | Baihuamai | 90 | Keyi 23 |
| 91 | Canzhouxiaomai | 91 | Jimai 20 | 91 | Baimangmai | 91 | Nongda 139 |
| 92 | Gaoyou 504 | 92 | Jimai 21 | 92 | Jiangmai | 92 | Nongda 311 |
| 93 | Han 4589 | 93 | Jimai 22 | 93 | Yuqiumai | 93 | Pingliang 30 |
| 94 | Handan 6050 | 94 | Jimai 4 | 94 | Hongmangzi | 94 | Gongnong 4 |
| 95 | Heimangmai | 95 | Jinan 10 | 95 | Hanzhongbai | 95 | Jinmai 8 |
| 96 | Heng 5229 | 96 | Jinan 13 | 96 | Hongxumai | 96 | Jinzhong 103 |
| 97 | Heng 7228 | 97 | Jinan 2 | 97 | Suotiaohongmai | 97 | Taifu 1 |
| 98 | Heng95 guan26 | 98 | Jining 3 | 98 | Xiaosanyuehuang | 98 | Beijing 15 |
| 99 | Hengguan 35 | 99 | Han 4589 | 99 | Baimaizi | 99 | Fengkang 2 |
| 100 | Hengmai 2 | 100 | Heng 136 | 100 | Chengduguangtou | 100 | Fengkang 8 |
| 101 | Hengshui 6404 | 101 | Jimai 6 | 101 | Honghuamai | 101 | Kecheng 1 |
| 102 | Hengyou 18 | 102 | Luomai 22 | 102 | Huanxiangguo | 102 | Pingliang 32 |
| 103 | Hulutou | 103 | Qingmai 7 | 103 | Tongjiabaxiaomai | 103 | Jimai 17 |
| 104 | Ji 92 - 5203 | 104 | Xinong 1043 | 104 | Yangmai | 104 | Tang 78042 |
| 105 | Jimai 10 | 105 | Hongliang 4 | 105 | Zipi | 105 | Yanan 11 |
| 106 | Jimai 18 | 106 | Jinmai 16 | 106 | Biantouguangkemai | 106 | Yanan 18 |
| 107 | Jimai 22 | 107 | Jinmai 25 | 107 | Changmangshibiantou | 107 | Jinmai 11 |
| 108 | Jimai 26 | 108 | Yunhan 22-33 | 108 | Dianxihongkeyangmai | 108 | Jinmai 16 |
| 109 | Jimai 29 | 109 | An 86 Zhong 17 | 109 | Yangmai | 109 | Lvhan 328 |
| 110 | Jimai 30 | 110 | Bawangbian | 110 | Zhugoumai | 110 | Taiyuan 2112 |
| 111 | Jimai 32 | 111 | Baicaomai | 111 | Zhushimai | 111 | An 85 Zhong 124-1 |
| 112 | Jimai 41 | 112 | Baiqimai | 112 | Baidongmai | 112 | Beijing 8694 |
| 113 | Jimai 6 | 113 | Baitutou | 113 | Chunmai | 113 | Beinongda BL8 |
| 114 | Jimai 9 | 114 | Baolin 9 | 114 | Hongchunmai | 114 | Hebuyu 6068 |
| 115 | Jimaiyihao | 115 | Baomai 5 | 115 | Hongchunmai | 115 | Jinghua 1 |
| 116 | Shi 4185 | 116 | Beijing 837 | 116 | Hongdongmai | 116 | Jingnong 81-49 |
| 117 | Shijiazhuang 407 | 117 | Beijing 8686 | 117 | Hongdongmai | 117 | Jingnong 86-89 |
| 118 | Shijiazhuang 8 | 118 | Beijing 8694 | 118 | Hongdongmai | 118 | Jingpin 12 |
| 119 | Shimai 12 | 119 | Beinong 2 | 119 | Hongjinbaoyin | 119 | Pinkang 244 |
| 120 | Shimai 13 | 120 | Bima 1 | 120 | Wumangchunmai | 120 | Qiaoliang BW41 |
| 121 | Silenghonghulutou | 121 | Cangmai 6001 | 121 | Wumangchunmai | 121 | Xiaoshan 8 |
| 122 | Shite 14 | 122 | Cangmai 6005 | 122 | Buyanghong | 122 | Yuandong 821 |
| 123 | Baicaomai | 123 | Cangzhouxiaomai | 123 | Baiqimai | 123 | Yuandong 822 |
| 124 | Bainong 3217 | 124 | Changle 5 | 124 | Huomai | 124 | Zhongda 89-60192-2 |
| 125 | Luohan 2 | 125 | Chang 4640 | 125 | Baimangmai | 125 | Qingshui 15-41(2) |
| 126 | Luohan 3 | 126 | Chang 4738 | 126 | Baitiaoyu | 126 | Xifeng 16 |
| 127 | Luohan 6 | 127 | Chang 4853 | 127 | Chushanbao | 127 | Lang 8302 |
| 128 | Luomai 9769 | 128 | Chang 5259 | 128 | Dakoumai | 128 | Tang 85-5032 |
| 129 | Luoyang 8628 | 129 | Chang 6154 | 129 | Fumai | 129 | Changzhi 5557 |
| 130 | Luoyang 9048 | 130 | Chang 6359 | 130 | Honggoudou | 130 | Changzhi 6406 |
| 131 | Wenmai 6 (Yumai 49) | 131 | Chang 6452 | 131 | Hongheshangtou | 131 | Jin 3052-5 |
| 132 | Yanzhanyihao | 132 | Chang 6794 | 132 | Huangguaxian | 132 | Jinmai 31 |
| 133 | Yumai 13 | 133 | Chang 6878 | 133 | Jiangmai | 133 | Jinghe 91-P19 |
| 134 | Yumai 18 | 134 | Chang 8744 | 134 | Pingyuan 50 | 134 | Jingnong 94-32 |
| 135 | Yumai 2 | 135 | Changmai 6135 | 135 | Sanyuehuang | 135 | Pindong 904110-3 |
| 136 | Yumai 38 | 136 | Changwu 131 | 136 | Youmangsaogudan | 136 | Zhongyou 9507 |
| 137 | Yumai 47 | 137 | Changwu 134 | 137 | Youzimai | 137 | Taiyuan 351 |
| 138 | Yumai 48 | 138 | Changwu 89(1)3-4 | 138 | Dayuhua | 138 | Jin 1410 |
| 139 | Yumai 8 | 139 | Changzhi 516 | 139 | Meiqianwu | 139 | Ganmai 6 |
| 140 | Zhengzhou 24 | 140 | Changzhi 620 | 140 | Baibiansui | 140 | Dingxi 24 |
| 141 | Zhoumai 18 | 141 | Dan R8043 | 141 | Baituzitou | 141 | Ganmai 46 |
| 142 | Aiganzao | 142 | Dan R8093 | 142 | Banjiemang | 142 | Ganmai 8 |
| 143 | Huaimai 18 | 143 | Dan R8108 | 143 | Dalibanmang | 143 | Hongtu |
| 144 | Huaishu 10 | 144 | Dan R8194 | 144 | Laolaixia | 144 | Huzhuhong |
| 145 | Xuzhou 21 | 145 | Dongxie 2 | 145 | Louguding | 145 | Xiangnong 3 |
| 146 | Xuzhou 6 | 146 | Fengkang 13 | 146 | Xishanbiansui | 146 | Huining 10 |
| 147 | Ningchun 4 | 147 | Hanxuan 10 | 147 | Zijiehong | 147 | Huining 5 |
| 148 | Luonong 10 | 148 | Hanxuan 11 | 148 | Baimangmai | 148 | Jinmai 303 |
| 149 | Mazhamai | 149 | Hanxuan 12 | 149 | Hongqiangchang | 149 | Jinmai 4 |
| 150 | Baolin 9 | 150 | Hanxuan 1 | 150 | Laoqimai | 150 | Linnong 12 |
| 151 | Baomai 5 | 151 | Hanxuan 2 | 151 | Mazhamai | 151 | Longchun 7 |
| 152 | Bima 1 | 152 | Hanxuan 3 | 152 | Qiangchangmai | 152 | Qingfeng 1 |
| 153 | Changwu 131 | 153 | Heimangmai | 153 | Tumangmai | 153 | Wuchun 1 |
| 154 | Changwu 89 (1) 3 - 4 | 154 | Hongheshang | 154 | Baihuomai | 154 | Wudu 5 |
| 155 | Dali 1 | 155 | Hulutou | 155 | Sanyuehuang | 155 | Xifeng 10 |
| 156 | Dali 52 | 156 | Huapei 6 | 156 | Xiaofoshou | 156 | Xifeng 9 |
| 157 | Fengchan 1 | 157 | Huabei 187 | 157 | Zhongguochun | 157 | Zhangchun 9 |
| 158 | Fengchan 3 | 158 | Ji 92-5203 |  |  | 158 | Ningchun 4 |
| 159 | Fuzhuang 30 | 159 | Jimai 10 |  |  | 159 | Gaoyuan 338 |
| 160 | Jinguang | 160 | Jimai 22 |  |  | 160 | Gaoyuan 506 |
| 161 | Qinmai 3 | 161 | Jimai 26 |  |  | 161 | Qingchun 25 |
| 162 | Qinmai 7 | 162 | Jimai 29 |  |  | 162 | Qingchun 28 |
| 163 | Qingchun 1 | 163 | Jimai 2 |  |  | 163 | Gan 8221-1-1 |
| 164 | Qingchun 2 | 164 | jimai 30 |  |  | 164 | Gan 8358-2 |
| 165 | Shaanyou 225-9 | 165 | Jimai 32 |  |  | 165 | Gan Tal21-10-2 |
| 166 | Shaan 229 | 166 | Jimai 38 |  |  | 166 | Ning 87N2801 |
| 167 | Shaanhan 8675 | 167 | Jimai 41 |  |  | 167 | Shi 886 |
| 168 | Shaanhe 6 | 168 | Jimai 6 |  |  | 168 | Gaoyuan 602 |
| 169 | Shaannong 1 | 169 | Jimai 9 |  |  | 169 | Longdong 1 |
| 170 | Shaannong 2 | 170 | Jimai 1 |  |  | 170 | Jian 72 |
| 171 | Shaannong 7859 | 171 | Jishen 5099 |  |  | 171 | Rikaze 7 |
| 172 | Shaanyou 225 | 172 | Jian 26 |  |  | 172 | Rikaze 8 |
| 173 | Shaanzi 1869 | 173 | Jinguang |  |  | 173 | Rikaze 54 |
| 174 | Shuangfengshou | 174 | Jin 2148-7 |  |  | 174 | Zangdong 4 |
| 175 | Weimai 4 | 175 | Jinmai 13 |  |  | 175 | Taizhong 23 |
| 176 | Xi'an 8 | 176 | Jinmai 17 |  |  | 176 | Dixiuzao |
| 177 | Xinong 1043 | 177 | Jinmai 33 |  |  | 177 | Kangxiu 10 |
| 178 | Xinong 6028 | 178 | Jinmai 39 |  |  | 178 | Yuanshan |
| 179 | Xinong 928 | 179 | Jinmai 44 |  |  | 179 | Jinmai 2148 |
| 180 | Xinong 979 | 180 | Jinmai 47 |  |  | 180 | Longxi 35 |
| 181 | Yanan 15 | 181 | Jinmai 50 |  |  | 181 | Fufan 904 |
| 182 | AimengniuⅣxing | 182 | Jinmai 51 |  |  | 182 | Taizhongxuan 2 |
| 183 | Baitutou | 183 | Jinmai 53 |  |  | 183 | Hechang 45 |
| 184 | Changle 5 | 184 | Jinmai 54 |  |  | 184 | Shuwan 8 |
| 185 | Jimai 21 | 185 | Jinmai 57 |  |  | 185 | Sichuan 51 |
| 186 | Jinan 10 | 186 | Jinmai 63 |  |  | 186 | Chuanmai 10 |
| 187 | Jinan 13 | 187 | Jinmai 68 |  |  | 187 | Fan 6 |
| 188 | Jinan 2 | 188 | Jinmai 72 |  |  | 188 | Xichangfanxiumai |
| 189 | Jining 3 | 189 | Jinmai 79 |  |  | 189 | Yaanzao |
| 190 | Lu 215953 | 190 | Jinmai 91 |  |  | 190 | Fengmai 11 |
| 191 | Lude 1 | 191 | Jinnong 207 |  |  | 191 | Bimai 26 |
| 192 | Lumai 14 | 192 | Jintai 102 |  |  | 192 | Chuan 7911 |
| 193 | Lumai 15 | 193 | Jintai 114 |  |  | 193 | Chuanmai 19 |
| 194 | Lumai 17 | 194 | Jintai 1310 |  |  | 194 | Hongai 1 |
| 195 | Lumai19 | 195 | Jintai 182 |  |  | 195 | Mianyang 11 |
| 196 | Lumai 1 (Aimengniu) | 196 | Jing 411 |  |  | 196 | Xichang 5548-9 |
| 197 | Lumai 21 | 197 | Jingdong 82 Dong 307 |  |  | 197 | Nanyuan 1 |
| 198 | Lumai 23 | 198 | Jingdong 83 Dong 65 |  |  | 198 | Pu 170 |
| 199 | Lumai 3 | 199 | Jingdong 8 |  |  | 199 | Yunmai 28 |
| 200 | Lumai 5 | 200 | Jinghe 8922 |  |  | 200 | Yunmai 29 |
| 201 | Lumai 8 | 201 | Jinghua 1 |  |  | 201 | Bimai 10 |
| 202 | Shannongfu 63 | 202 | Jingnong 79-15 |  |  | 202 | Bimai 13 |
| 203 | Taishan 23 | 203 | Jingnong 80 Jian 107 |  |  | 203 | Guinong 10 |
| 204 | Taishan 24 | 204 | Jingnong 84-6786 |  |  | 204 | Hemai 8052 |
| 205 | Yan 881414 | 205 | Jingpin 11 |  |  | 205 | Kenguia 1 |
| 206 | Yannong 21 | 206 | Jingpin 30 |  |  | 206 | Pan 86001-3 |
| 207 | Xiaoyan 22 | 207 | Jingpin 3 |  |  | 207 | Pan 88080-3-1-1 |
| 208 | Xiaobaimai | 208 | Jingshuang 16 |  |  | 208 | Chuan 83C-1001 |
| 209 | Chang 4640 | 209 | Jingshuang 2 |  |  | 209 | Chuanmai 22 |
| 210 | Chang 4738 | 210 | Jingxuan 20 |  |  | 210 | Chuanyu 12 |
| 211 | Chang 6154 | 211 | Jingxuan 25 |  |  | 211 | Dian 8613 |
| 212 | Chang 6359 | 212 | Jingyan 85 Jian 28 |  |  | 212 | Jingmai 2 |
| 213 | Chang 6452 | 213 | Kenong 199 |  |  | 213 | Yunmai 33 |
| 214 | Chang 6878 | 214 | Keyi 26 |  |  | 214 | Yunmai 34 |
| 215 | Changzhi 516 | 215 | Keyi 29 |  |  | 215 | Gui 775 |
| 216 | Changzhi 620 | 216 | Lin 138 |  |  | 216 | Guinong Y13 |
| 217 | Hanxuan 11 | 217 | Linfen 8050 |  |  | 217 | Xingyi 4 |
| 218 | Hanxuan 12 | 218 | Linfeng 3 |  |  | 218 | Xingzhuai 3 |
| 219 | Hanxuan 1 | 219 | Linfeng 518 |  |  | 219 | Mianyang 26 |
| 220 | Hanxuan 2 | 220 | Linhan 5089 |  |  | 220 | Qianjian 28 |
| 221 | Hongheshang | 221 | Linhan 5367 |  |  | 221 | Xingyingwu 3 |
| 222 | Jinmai 13 | 222 | Linhan 6105 |  |  | 222 | Dian 622-525-2 |
| 223 | Jinmai 16 | 223 | Linhan 6 |  |  | 223 | Kashibaipi |
| 224 | Jinmai 17 | 224 | Linhan 917 |  |  | 224 | Tuokexun 1 |
| 225 | Jinmai 25 | 225 | Linhan 935 |  |  | 225 | Xindong 2 |
| 226 | Jinmai 39 | 226 | Linkang 5108 |  |  | 226 | Kashi 1 |
| 227 | Jinmai 44 | 227 | Longjian 196 |  |  | 227 | Yinong 8 |
| 228 | Jinmai 47 | 228 | Longjian 294 |  |  | 228 | Changdong 5 |
| 229 | Jinmai 50 | 229 | Lude 1 |  |  | 229 | Jiudong 2 |
| 230 | Jinmai 51 | 230 | Lumai 14 |  |  | 230 | Tuchun 6 |
| 231 | Jinmai 53 | 231 | Lumai 15 |  |  | 231 | Xinchun 2 |
| 232 | Jinmai 54 | 232 | Lumai 17 |  |  | 232 | 1817 |
| 233 | Jinmai 57 | 233 | Lumai 19 |  |  | 233 | Shite 14 |
| 234 | Jinmai 5 | 234 | Lumai 23 |  |  | 234 | Bima 1 |
| 235 | Jinmai 63 | 235 | Lumai 3 |  |  | 235 | Bima 4 |
| 236 | Jinmai 68 | 236 | Lumai 5 |  |  | 236 | Jingyang 60 |
| 237 | Jinmai 72 | 237 | Lumai 8 |  |  | 237 | Qida 195 |
| 238 | Jinmai 79 | 238 | Lunkang 7 |  |  | 238 | Shijiazhuang 4 |
| 239 | Jinnong 207 | 239 | Lunxuan 987 |  |  | 239 | Shijiazhuang 407 |
| 240 | Jintai 170 | 240 | Mazhamai |  |  | 240 | Neixiang 5 |
| 241 | Linfeng 3 (Linhan 536) | 241 | Mingxian 169 |  |  | 241 | Jingyang 30 |
| 242 | Linfeng 518 | 242 | Ningdong 11 |  |  | 242 | Shannong 9 |
| 243 | Linfeng 615 | 243 | Nongda 135 |  |  | 243 | Xinong 6028 |
| 244 | Linhan 6105 | 244 | Nongda 146 |  |  | 244 | Jinan 2 |
| 245 | Linhan 6 | 245 | Nongda 155 |  |  | 245 | Hengshui 7004 |
| 246 | Linhan 917 | 246 | Nongda 183 |  |  | 246 | Shijiazhuang 34 |
| 247 | Linhan 935 | 247 | Nongda 20074 |  |  | 247 | Shijiazhuang 54 |
| 248 | Linkang 5108 | 248 | Nongda 311 |  |  | 248 | Zaoxinshi |
| 249 | Mingxian 169 | 249 | Nongda 3195 |  |  | 249 | Anxuan 2 |
| 250 | Pingyang 348 | 250 | Nongda 33 |  |  | 250 | Yuanzhu 55 |
| 251 | Taiyuan 633 | 251 | Nongda 36 |  |  | 251 | Zhengzhou 4 |
| 252 | Yunhan 2028 | 252 | Nongda 81146 |  |  | 252 | Zhengzhou 6 |
| 253 | Yunhan 20410 | 253 | Pingliang 35 |  |  | 253 | Fengchan 3 |
| 254 | Yunhan 21 - 30 | 254 | Pingyang 348 |  |  | 254 | Fuzhuang 30 |
| 255 | Yunhan 22 - 33 | 255 | Qinmai 3 |  |  | 255 | Jinguangmai |
| 256 | Yunhan 23 - 35 | 256 | Qinmai 7 |  |  | 256 | Xiannong 39 |
| 257 | PANDAS | 257 | Qingfeng 1 |  |  | 257 | Changwei 20 |
| 258 | SALGEMMA | 258 | Shannongfu 63 |  |  | 258 | Jinan 9 |
| 259 | Lovrin 10 | 259 | Shannongyoumai 2 |  |  | 259 | Yannong 3 |
| 260 | Triumph | 260 | Shanyou 2 |  |  | 260 | Youbao |
| 261 | Early Premium | 261 | Shan225-9 |  |  | 261 | Xiaonong 76189 |
| 262 | TAM-110 | 262 | Shaan 229 |  |  | 262 | Zhangdong 29 |
|  |  | 263 | Shaanhan 8675 |  |  | 263 | 12040 |
|  |  | 264 | Shaanhe 6 |  |  | 264 | Cangzhou 1 |
|  |  | 265 | Shaannong 1 |  |  | 265 | Hengdalihong |
|  |  | 266 | Shaannong 2 |  |  | 266 | Jimai 14 |
|  |  | 267 | Triumph |  |  | 267 | Jimai 20 |
|  |  | 268 | Shuangfengshou |  |  | 268 | Pin 39 |
|  |  | 269 | Shunmai 1718 |  |  | 269 | Bainong 3217 |
|  |  | 270 | Silenghonghulutou |  |  | 270 | Bonong 7023 |
|  |  | 271 | Tai 13606 |  |  | 271 | Mengxian 2 |
|  |  | 272 | Tai 712 |  |  | 272 | Wan 7107 |
|  |  | 273 | Taiyuan 566 |  |  | 273 | Yu 7106-0-22-1-3-2B |
|  |  | 274 | Taiyuan 633 |  |  | 274 | Yumai 2 |
|  |  | 275 | Taishan 23 |  |  | 275 | Zhengzhou 741 |
|  |  | 276 | Taishan 24 |  |  | 276 | Huaimai 11 |
|  |  | 277 | Weimai 4 |  |  | 277 | Ningfengmai |
|  |  | 278 | Xifeng 16 |  |  | 278 | Aifeng 3 |
|  |  | 279 | Xifeng 20 |  |  | 279 | Baomai 3 |
|  |  | 280 | Xifeng 9 |  |  | 280 | Baomai 5 |
|  |  | 281 | Xiaobaimai |  |  | 281 | Shan 70-1 |
|  |  | 282 | Xiaoshan 8 |  |  | 282 | Shan 7219-8-11-2-1 |
|  |  | 283 | Xindong 20 |  |  | 283 | Xiannong 151 |
|  |  | 284 | Xindong 22 |  |  | 284 | Xiaoyan 4 |
|  |  | 285 | Yannong 19 |  |  | 285 | Xiaoyan 6 |
|  |  | 286 | Yannong 21 |  |  | 286 | Lainongpinxi 22 |
|  |  | 287 | Yanan 15 |  |  | 287 | Lumai 1 |
|  |  | 288 | Yanda 1817 |  |  | 288 | Lunong 784081 |
|  |  | 289 | Yuandong 3 |  |  | 289 | Taishan 1 |
|  |  | 290 | Yuandong 834 |  |  | 290 | Taishan 4 |
|  |  | 291 | Yuandong 847 |  |  | 291 | Taishan 7 |
|  |  | 292 | Yuandong 856 |  |  | 292 | Yannong 15 |
|  |  | 293 | Yunhan 102 |  |  | 293 | Jinmai 20 |
|  |  | 294 | Yunhan 115 |  |  | 294 | Bu 84111 |
|  |  | 295 | Yunhan 2028 |  |  | 295 | Wo 80 |
|  |  | 296 | Yunhan 20410 |  |  | 296 | Pingliang 35 |
|  |  | 297 | Yunhan 21-30 |  |  | 297 | Jimai 19 |
|  |  | 298 | Yunhan 23-35 |  |  | 298 | Jimai 23 |
|  |  | 299 | Yunhan 618 |  |  | 299 | Jishi 5032 |
|  |  | 300 | Yunhan 719 |  |  | 300 | Shi 82-5201 |
|  |  | 301 | Yunhan 805 |  |  | 301 | Hua 852895-2 |
|  |  | 302 | Zaosui 21 |  |  | 302 | Yu 30691-1-3 |
|  |  | 303 | Zaosui 65 |  |  | 303 | Yumai 14 |
|  |  | 304 | Zaosui 66 |  |  | 304 | Yumai 18 |
|  |  | 305 | Early premium |  |  | 305 | Yumai 7 |
|  |  | 306 | Zhangdong 29 |  |  | 306 | Huaimai 12 |
|  |  | 307 | Zhengfeng 9962 |  |  | 307 | Xuzhou 19 |
|  |  | 308 | Zhengzhou 24 |  |  | 308 | Xuzhou 21 |
|  |  | 309 | Zhong 7902 |  |  | 309 | Qinmai 3 |
|  |  | 310 | Zhong 86 I-50455 |  |  | 310 | Qinmai 8 |
|  |  | 311 | Zhongda 86-Jian 2 |  |  | 311 | Shannong 7859 |
|  |  | 312 | Zhongda 91-Pin 9 |  |  | 312 | Lumai 10 |
|  |  | 313 | Zhongda 92-Jian 49 |  |  | 313 | Lumai 5 |
|  |  | 314 | Zhongda 92-Pin 8 |  |  | 314 | Lumai 7 |
|  |  | 315 | Zhonghan 110 |  |  | 315 | Lumai 9 |
|  |  | 316 | Zhongmai 175 |  |  | 316 | Lunong 86(5)174 |
|  |  | 317 | Zhongmai 9 |  |  | 317 | Luzi 0863169 |
|  |  | 318 | Zhongsu 68 |  |  | 318 | Luzi 0884142 |
|  |  | 319 | Zhongyin 6 |  |  | 319 | Shannong PH85-4 |
|  |  | 320 | Zhongyou 9507 |  |  | 320 | Teng 80-1-2 |
|  |  | 321 | Zhongzuo 60064 |  |  | 321 | Yanzhong 144 |
|  |  | 322 | Zhongzuo 60115 |  |  | 322 | Jin 865096 |
|  |  | 323 | Ziganbaimangxian |  |  | 323 | Jinmai 37 |
|  |  |  |  |  |  | 324 | Linyuan 129 |
|  |  |  |  |  |  | 325 | Pingyang 27 |
|  |  |  |  |  |  | 326 | Han 4564 |
|  |  |  |  |  |  | 327 | Ji 93C6 156-2 |
|  |  |  |  |  |  | 328 | Anmai 95 Zhong 35 |
|  |  |  |  |  |  | 329 | Lankao 906 |
|  |  |  |  |  |  | 330 | Wenmai 6 |
|  |  |  |  |  |  | 331 | Yumai 54 |
|  |  |  |  |  |  | 332 | Zheng 87305-0-13 |
|  |  |  |  |  |  | 333 | Zhengzhou 8761 |
|  |  |  |  |  |  | 334 | Zhengzi R84019-0-7-4-0-1 |
|  |  |  |  |  |  | 335 | Xuzhou 22 |
|  |  |  |  |  |  | 336 | Fen 22 |
|  |  |  |  |  |  | 337 | Gaoyou 503 |
|  |  |  |  |  |  | 338 | Liken 2 |
|  |  |  |  |  |  | 339 | Shannong 229 |
|  |  |  |  |  |  | 340 | Jinan 17 |
|  |  |  |  |  |  | 341 | Laizhou 953 |
|  |  |  |  |  |  | 342 | Lumai 22 |
|  |  |  |  |  |  | 343 | Gaobi-13 |
|  |  |  |  |  |  | 344 | Shan 8786-0-3 |
|  |  |  |  |  |  | 345 | Huangtai 103 |
|  |  |  |  |  |  | 346 | Jimai 19 |
|  |  |  |  |  |  | 347 | Jinyang 1045 |
|  |  |  |  |  |  | 348 | Maijin 1 |
